# Evaluating Process and Outcomes of Public Involvement in Applied Health and Social Care Research: A Rapid Systematic Review

**DOI:** 10.1111/hex.70160

**Published:** 2025-01-22

**Authors:** Angela Wearn, Kerry Brennan‐Tovey, Emma A. Adams, Hayley Alderson, Judy Baariu, Mandy Cheetham, Victoria Bartle, Lucy Palfreyman, Violet Rook, Felicity Shenton, Sheena E. Ramsay, Eileen Kaner

**Affiliations:** ^1^ Population Health Sciences Institute, Faculty of Medical Sciences Newcastle University Newcastle Upon Tyne UK; ^2^ Department of Nursing, Midwifery and Health Northumbria University Newcastle Upon Tyne UK; ^3^ NIHR Applied Research Collaboration North East North Cumbria Public Advisory Network Newcastle Upon Tyne UK; ^4^ Cumbria, Northumberland, Tyne and Wsupear NHS Foundation Trust, St Nicolas' Hospital Newcastle Upon Tyne UK

**Keywords:** coproduction, evidence synthesis, public involvement, research, systematic review

## Abstract

**Objective:**

Public Involvement (PI) in applied health and social care research has grown exponentially in the UK. This review aims to synthesise published UK evidence that evaluates the process and/or outcome(s) of PI in applied health and social care research to identify key contextual factors, effective strategies, outcomes and public partner experiences underpinning meaningful PI in research.

**Methods:**

Following a pre‐registered protocol, we systematically searched four databases and two key journals for studies conducted within the UK between January 2006 and July 2024. A team of public partners and researchers carried out independent dual screening and data extraction. Included studies were narratively synthesised via Framework Synthesis.

**Results:**

Nineteen studies evaluated the PI process with a range of populations including National Health Service (NHS) users, carers, and low‐income communities. No specific outcome evaluations were identified. Through their experience, public partners described important components of meaningful PI such as mutual respect and seeing and contributing to change, as well as some unintended harms of involvement. Harms related to ‘experiencing negative attitudes’, ‘emotional burden of involvement’, ‘frustration and disappointment’ and ‘further marginalisation’. Meaningful PI was underpinned by structural, organisational, interpersonal and individual factors; as well as practical and principle‐based strategies of involvement. Both public partners and researchers reflected on a range of outcomes of meaningful PI including changes to the research process and longer term impacts on organisations, researchers and public partners.

**Conclusions:**

PI in research must be facilitated at multiple levels to reduce unintended harm and encourage meaningful and impactful outcomes. Findings are summarised within a model which gives an overview of priorities for individual researchers, organisations and funders to ensure best practice is achievable. From a methodological perspective, researchers should prioritise robust, transparent and co‐produced approaches to evaluating PI to increase knowledge in the field.

**Patient and Public Involvement:**

A regional public advisory network provided insight on the relevance and acceptability of the review concept. Our core research team included three public partners. Public partners contributed to the development of the initial review protocol, abstract and full‐text screening, reviewing findings and their interpretation and writing the final report.

## 1 Background

Public involvement (PI) in research refers to an active bidirectional partnership between researchers and patients, carers and other members of the public to influence research processes (e.g. priority‐setting, development and delivery of projects) and/or research outcomes. This definition is in line with Arnstein's ‘ladder of participation’ [1], which is widely referred to when highlighting the spectrum of ways in which members of the public can be involved in research [2]. Broadly, this spectrum ranges from non‐participation (e.g. manipulation) and tokenism (e.g. informing, consulting or placating public partners) to more meaningful forms of involvement, focused on partnership working and an increased ability to influence decision‐making and allocation of resources.

From its creation in 2006, the National Institute for Health and Care Research (NIHR) has developed policies to support and promote PI in research [3]. Improving the nation's health and wellbeing through PI is a major focus across research funders, research institutions, local government, charity organisations and the National Health Service (NHS) [2, 4]. Many research funders within the UK have increasingly encouraged, and now mandate, PI in applied health and social care research funding applications (i.e. applied research being that which aims to identify practical solutions or improvement to community‐identified problems).

Systematic reviews have demonstrated that active involvement of the public has benefits for health and social care research [5, 6]. For example, PI contributes to the development of user‐focused research objectives, user‐friendly information, more appropriate recruitment strategies, consumer‐focused data interpretation and enhanced implementation and dissemination of study results [5]. In addition, there are reported positive impacts for members of the public (increased confidence, increased life skills), researchers (increased rapport with communities) and communities (increased awareness and knowledge about their health condition) [6].

However, much current evidence is based on researchers' reflections on PI. Little is known about the extent to which transparent and robust evidence is used to evaluate PI in applied health research. Researchers have previously called for greater focus on the reporting of context within the evaluation of PI, to strengthen understanding of factors that help or hinder the likelihood of positive outcomes [5, 7]. This is particularly important to minimise negative unintended consequences of PI such as time and financial burdens and lack of clarity around expectations [8].

Synthesising existing evidence which specifically evaluates the process and/or outcomes of PI in applied health and social care research, inclusive of public partner experiences and perspectives, would therefore be of benefit to facilitate high‐quality PI, demonstrate evidence‐based benefits of partnership working, and facilitate a deeper understanding of the links between contextual factors, effective PI strategies and outcomes. These areas can also help clarify current gaps in evidence and identify priorities for future evaluations.

We aimed to synthesise published literature that evaluated the process and/or outcomes of PI within applied health and social care research, in line with the following questions:

1. What strategies and contextual factors facilitate PI in health and social care research?
2. What are the reported outcomes of PI in health and social care research?
3. What are the reported experiences of public partners, involved in the design and/or delivery of health and social care research?

## 2 Method

This review was conducted in line with PRISMA [9] and Cochrane Rapid Review Guidance [10]. A protocol for the review was registered prior to the review taking place on the PROSPERO database [CRD42022310210]. Despite including both qualitative and quantitative studies in our review, all but one of the final included studies were qualitative. As such, we adhered to ENTREQ guidelines for reporting qualitative synthesis [11]. PI was reported in line with GRIPP2 [12] guidance (see Supporting Information A).

### 2.1 Search Strategy

A comprehensive search of EMBASE, MEDLINE, CINAHL and ASSIA databases was conducted. We included literature published between January 2006 and July 2024 to align with the establishment of the NIHR in 2006, which marked a significant advancement in UK health research funding and development of PPI policies and practice. Handsearching was also conducted in key journals in the field (Health Expectations and BMC Research Involvement and Engagement). Our search strategy was developed in consultation with an Information Specialist, an example strategy (EMBASE) is presented in Supporting Information B. The reference lists of all included full‐text articles were hand‐searched for additional eligible literature. Forward citation of included articles was also conducted. Initial searches were conducted from January to February 2023 with updated hand‐ and citation‐searching carried out in July 2024 to incorporate any relevant literature published between February 2023 and July 2024.

### 2.2 Inclusion and Exclusion Criteria

We included original research articles that evaluated the process and/or outcomes of PI within applied health and social care research. Applied research in this context refers to that which aims to tackle community‐identified problems and improve health and social care outcomes. Papers evaluating PI in clinical research (e.g. controlled drug trials) were beyond the scope for this review and therefore excluded (see Supporting Information C). Outcomes, in this instance, relate to changes, outputs, events or impacts that occurred either directly or indirectly as a result of PI. Given we are considering PI within the context of UK research guidance and requirements, included studies were limited to those conducted within the UK. In addition, articles were excluded if no analysis of primary data was included and/or if the full‐text was not available in English. Further details on exclusion and inclusion criteria, with justification, is presented in Supporting Information C.

### 2.3 Screening and Data Extraction

Identified records were initially stored in EndNote. Following de‐duplication, a pilot screening exercise was conducted across the screening team. Independent dual screening of title and abstracts was then performed via Rayyan (www.rayyan.ai). Rates of concordance were high (99.2%). Conflicts were resolved via a third reviewer. The remaining full‐text articles were recorded in a standardised Excel form (which had been piloted with 5 articles). Full‐text articles were again dual‐screened for inclusion with a third reviewer resolving any conflicts. The lead author (A.W.) extracted key data using a piloted form. A second reviewer independently checked extracted data alongside full texts for completeness.

### 2.4 Quality Assessment

#### 2.4.1 Methodological Quality of Studies

The Critical Appraisal Skills Programme (CASP) checklist [13] was used to assess the methodological quality of the evaluation papers. This checklist is regularly used within health and social care‐related literature reviews [14] and prompts reviewers to appraise quality based upon 10 questions relating to the reporting of the study aims, appropriateness of methodology, research design and recruitment strategy and rigour of data analysis. An overall appraisal of value to the review is made for the final question (i.e. with higher quality papers scoring > 8; see Supporting Information E). Included studies were assessed by the lead author and checked by a second reviewer. Quality assessment was conducted for guidance purposes, with greater consideration given to papers of higher quality during theme development and interpretation [15, 16, 17].

#### 2.4.2 Quality of PI Reporting

The GRIPP2 short form was used to assess the quality of PI reporting [12]. The GRIPP2 is widely used as both a guide and a checklist, detailing five areas of importance when reporting PI to ensure transparency and increase the quality of the evidence base [12]. We assessed the quality of PI reporting in relation to both (1) *the original study being evaluated* and (2) *the evaluation study/process itself*. As above, this process was included for guidance purposes only, to provide insight on the level of detail included in published evaluations of PI.

### 2.5 Data Synthesis

Framework synthesis was used to synthesise studies [18]. In line with Thomas, O'Mara‐Eves, Harden and Newman [19] data synthesis was conducted in two stages: *Developing or selecting an initial framework* and *Recognising patterns through aggregation*.

#### 2.5.1 Selecting an Initial Framework/Model

**Figure 1:**
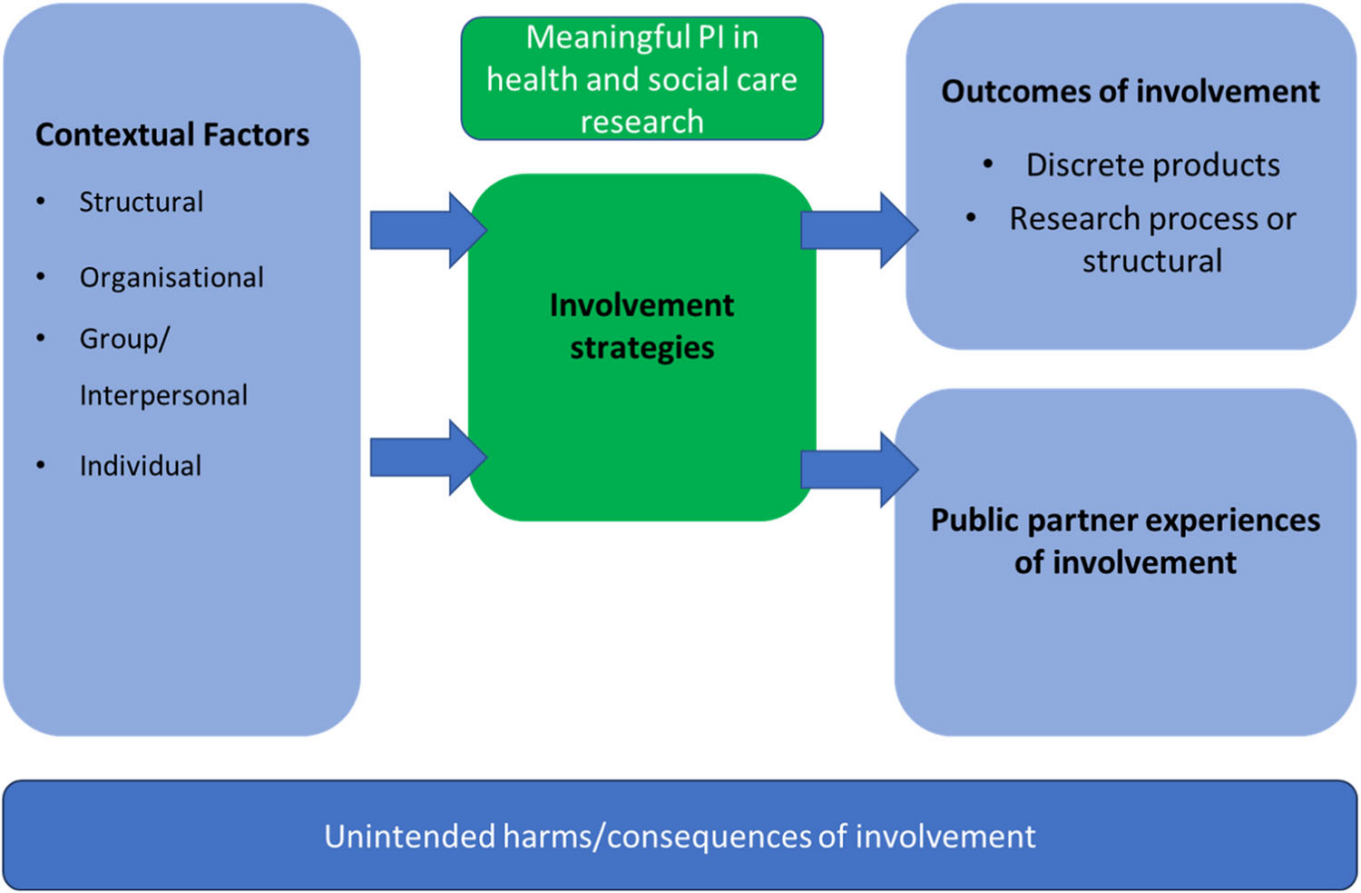
Initial coding framework.

Figure 1 shows our initial coding framework/model of meaningful PI in health and social care. The coding framework was informed by past literature [20, 21, 22] and through team discussions, particularly during the development of our research protocol.

#### 2.5.2 Recognising Patterns Through Aggregation

Text referring to the findings of included studies was line‐by‐line coded. This included both participant quotes and interpretative text from the author(s). Codes were grouped thematically in line with the initial model via Microsoft Excel, which was then amended and expanded iteratively throughout the analysis process. Coding and analysis were conducted by the lead author, who is an experienced qualitative researcher and PI professional. The coding and analysis were checked and discussed iteratively with the wider research team. Data/excerpts relating to each aspect of the model were extracted into tabular format and again considered in line with the overall aims of the review.

### 2.6 Patient and Public Involvement

The NIHR UK Standards for Public Involvement [23] were applied to support meaningful PI in this review (see Supporting Information D). The relevancy and acceptability of the review concept were discussed during a regional Public Advisory Network meeting which includes a diverse group of public members and small community organisations interested in shaping health and social care research. Following favourable opinion to move ahead with the review, three experienced public partners (V.B., L.P., V.R.) expressed interest in joining the core team. We took a partnership approach, whereby members of the public were equal partners/co‐investigators, contributing to decision‐making and delivery of the project. The lead author (A.W.) developed and delivered bespoke training to public partners to support their involvement and was identified as a core point of contact. Training content included background to the present project, a brief introduction to literature reviews, rapid systematic review methodology and terms, and use of screening software (www.rayyan.ai) including a step‐by‐step ‘getting started’ guide to support the screening process. During the screening and selection stages, public partners were paired with an experienced researcher (E.A.A., H.A., M.C.) as an additional source of support and guidance. Overall, public partners contributed to the development of the review protocol, screening and selection process, reviewing findings and interpretation and writing of the final report. It is estimated that each public partner spent 16–20 h working on the review as part of the core team. All PI was remunerated in line with NIHR guidelines [24].

## 3 Results

**Figure 2:**
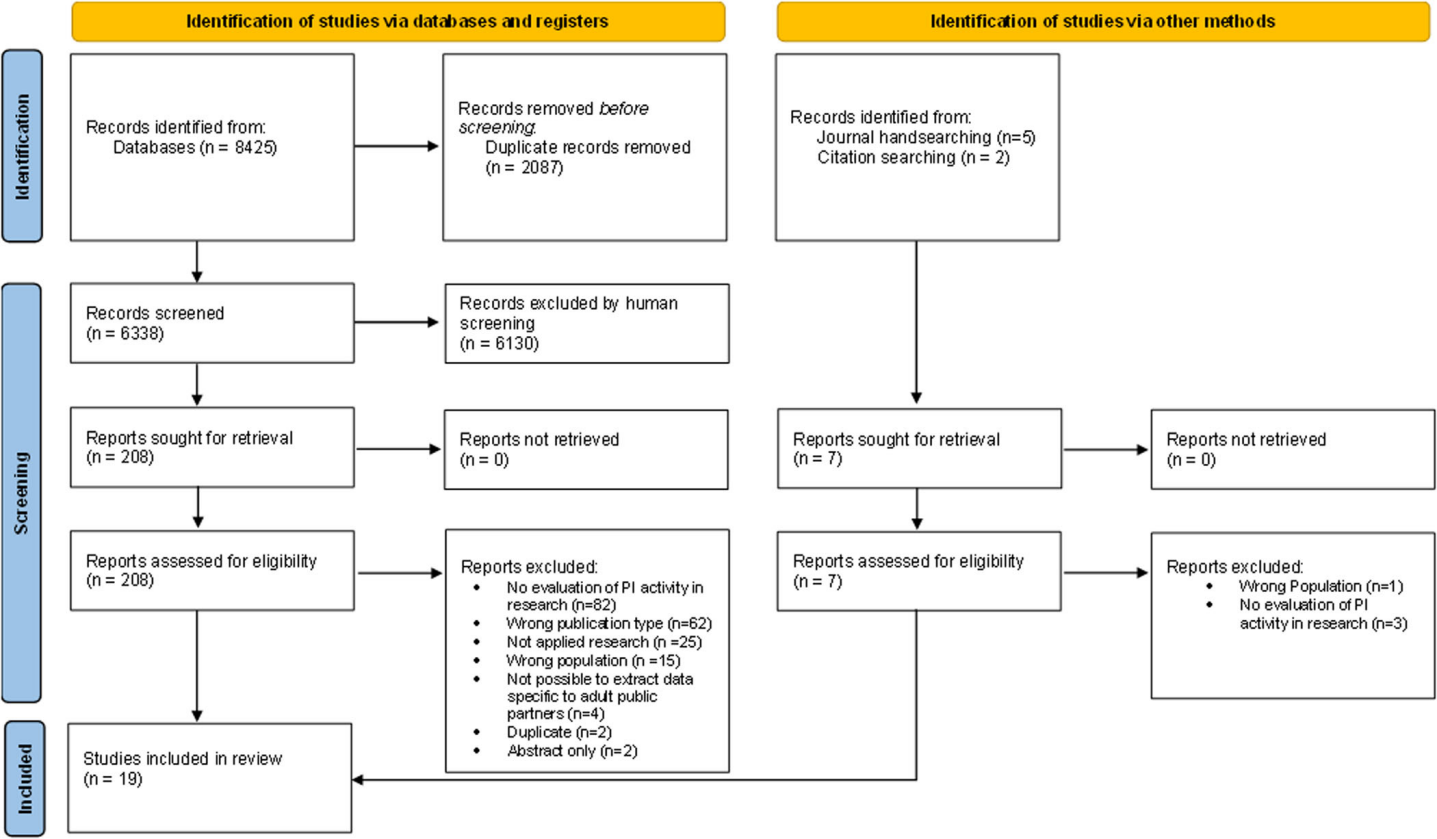
PRISMA flowchart.

Nineteen papers were included in the final review (see Figure 2).

### 3.1 Quality Assessment

#### 3.1.1 Methodological Quality of Included Papers

No papers were assessed as ‘low value’ overall; however, it was of note that over half (*n* = 10) lacked transparency/rigour in relation to data analysis procedures. Full ratings are available in Supporting Information E.

#### 3.1.2 Quality of PI Reporting

When referencing the original study being evaluated, quality of PI reporting was high – there were no studies deemed low quality. However, just under half (*n* = 9) of the included studies reported very little detail regarding how public partners were involved within the evaluation process itself beyond participation in e.g. focus groups (PI within the evaluation process referring to e.g. theme development/interpretation of findings, report writing or reviewing). Full ratings are available in Supporting Information F.

### 3.2 Summary of Study Characteristics

Study characteristics are summarised in Table 1. Almost all evaluations (*n* = 18) were qualitative in nature with only *n* = 1 being mixed methods inclusive of an online survey alongside semi‐structured interviews. No specific outcome evaluations were found during the literature search. All studies focused on the process of PI, with *n* = 6 of these mainly focused on public partner's experience. However, *n* = 12 studies did include reflections on a number of outcomes researchers and/or public partners felt came about as a result of PI.

**Table 1:** Summary of included studies evaluating public involvement in applied health and social care research.

		Original study (i.e. study being evaluated)			Evaluation study			
Author	Year	Topic area	Details of public partners	Role of public partners	Evaluation focus (relevant to this review)	Evaluation data collection methods	Evaluation data analysis methods	Public partner(s) contribution to evaluation process^a^
Aabe et al.	2019	Exploring support for Somali families who are affected by autism	1 Somali mother of an autistic child	Initiating project focus, co‐researcher across the research process.	Process and impact of co‐producing knowledge about autism within a UK Somali community.	Unclear ‐ reflective accounts from public partner	Mapping of first‐person narrative insights and impacts to existing theoretical models.	Providing reflections/feedback on experience, lead author on evaluation paper
Beighton et al.	2019	Effectiveness of annual health checks for adults with intellectual disabilities.	4 parent carers and 5 adults (aged 27‐40) with mild to moderate intellectual disabilities	Input on research design, data analysis and interpretation, dissemination and developing recommendations for further practice.	Experiences of public partners, with reflection on process and outcomes.	Focus Groups	Thematic analysis	Providing reflections/feedback on experience, sense‐checking initial analysis.
Brett et al.	2022	Impact of prostate cancer on men's health and wellbeing.	7 men who had experienced different stages of prostate cancer and treatment	To lead on a PPI workstream across the research process	Process and impact of public involvement within a large UK study.	Online survey and semi‐structured interviews	Descriptive analysis of survey data; Thematic analysis of interview data	Involvement in study design, development of materials, analysis of qualitative data and write up of paper.
Buffel	2019	Development of ‘age friendly’ communities.	18 Older adults (aged 58–74) from LGBTQ+ community.	Co‐researchers across the research process.	Experiences of co‐researchers, with reflection on process and outcomes.	Reflection meetings	Thematic analysis	Providing reflections/feedback on experience, review of key selected quotes during analysis.
Cotterell and Buffel	2023	Loneliness in ethically or sexually minoritised older people.	10 Older adults (age 50–79) from South Asian, Chinese or White British LGBT backgrounds.	Co‐researchers involved from reviewing research proposal to dissemination stages.	Experiences of co‐researchers, with reflection on process, benefits and challenges of involvement.	Focus Groups	Thematic analysis	Providing reflections/feedback during focus groups, reviewing researcher developed themes.
Devonport et al.	2018	Designing, testing and evaluating and intervention targeting emotional eating.	10 patients accessing weight management services/with binge‐eating disorder.	Validate research questions and inform research design and methods.	Process of public involvement in the development of obesity and binge‐eating research.	Post Participation Evaluation Questionnaire, Notes from group meetings, Written reflections from practitioner and researchers and written and verbal correspondence, consolidating sources 1–3.	Content analysis	Providing reflections/feedback questionnaire, verification of findings.
Evans et al.	2022	Evaluating models of care whereby general practitioners work in or alongside Emergency Departments in hospitals.	19 members of the public	Contributing to study management and delivery, steering committee representation and additional public input at stakeholder event.	Process and implementation of public involvement in a large scale, multi‐site study, with some reflections on outcomes.	Focus group interviews	Thematic analysis and review of public involvement logs	Involvement in development of materials, data collection and analysis, co‐authoring research paper.
Forbat et al.	2024	Provision of palliative and end of life care to older people living in care homes.	3 public partners with experience of care homes or palliative care.	Co‐researchers across the research cycle, from the conception of the study onwards.	Experiences of PI, with some reflections on outcomes/impacts.	One‐to‐one semi‐structured interview conducted by an independent researcher via telephone or video conferencing.	Thematic analysis	Providing reflections/feedback on experience, co‐development of interview topic guide, co‐authoring research paper.
Froggatt et al.	2016	Evaluating integrated working between care homes and primary care.	6 members of the public with prior personal or professional experience of care homes	Co‐researchers supporting in participant recruitment, interviews and data interpretation.	Process of public involvement	Group meetings and completion of reflective templates.	Unclear – data was coded and grouped into three dimensions of user involvement: methods, roles and outcomes.	Providing reflections/feedback on experience
Lithander et al.	2023	Improving the quality of life of people with Parkinson's.	3 public partners with personal experience of Parkinson's	Informing terminology, the development of patient‐facing materials and the recruitment process.	Reflections on outcomes and impacts of public involvement.	Public impact Logs. Further reflection as part of online meetings.	Unclear ‐ development of themes through online group meetings.	Providing reflections/feedback, theme development, co‐authoring evaluation paper.
Litherland et al.	2018	Improving the experience of dementia and enhancing active life.	4 people with dementia and 5 carers or former carers.	Shaping project materials, providing feedback on data collection methods, supporting data analysis and co‐presenting findings.	Process of involving dementia patients and carers in research, with reflections on public partner experiences and outcomes.	Focus Group and creation of a short film.	Not reported	Providing reflections/feedback on experience, Co‐authoring evaluation paper.
McMenamin et al.	2021	Evaluating an intervention for those with aphasia and exploring understandings of friendship.	2 female aphasia patients (aged 55+)	Collaboration across the research process.	Experiences of public partners with aphasia, with reflections on process.	Semi‐structured interviews	Thematic Analysis	Providing reflections/feedback on experience, involvement in data analysis and co‐authoring evaluation paper.
Rowe	2006	Experiences, needs and expectations of new parents to inform a Sure Start programme.	16 mothers of mostly pre‐school and primary school aged children.	Co‐researchers across the research process.	Experiences of parent researchers, with reflections on process and outcomes.	Postal questionnaires (pre and post study), Researcher diary (during data collection), focus group (after data collection)	Questionnaire data: collated and compared. Diary and focus group: Content analysis	Providing reflections and feedback on experiences.
Slade et al.	2016	Exploring ways to increase the extent to which mental health services promote recovery.	5 mental health service users and carers.	Participation within a Lived Experience Advisory Panel to review the research and provide feedback, advice and recommendations periodically over the programme.	Process and outcomes of working with a lived experience panel across a large 5 year programme of recovery research.	Collection of narrative reflections from public partners and wider research team.	Unclear ‐ narrative accounts were synthesised	Providing reflections and feedback on experiences, public partner as co‐author.
Stocker et al.	2021	Perceptions and experiences of primary care services for care home residents, relatives and professionals.	7 members of the public who have a relative residing in a care home, or are involved in working with/in care homes within a voluntary or professional capacity.	Involvement in qualitative data analysis.	Process of public involvement in qualitative data analysis with some reflection on outcomes.	Focus group	Thematic approach	Participation in a focus group.
Sutton and Weiss	2008	Exploring supplementary prescribing role(s) and practice.	10 NHS service users from a diabetes support group and chronic lung disease support group. Average age 60 years.	To provide advice on research design, preparation of research materials, data analysis and final report.	Process of involving those with a chronic condition in a research project exploring pharmacist supplementary prescribing.	Collation of meeting minutes, feedback from participants and notes taken by the researchers during the project time period.	Content analysis	Providing feedback to researchers.
Thomas et al.	2021	Poverty‐related mental distress	8 individuals living within low‐income communities.	Collaboration on shaping research questions and methods, data interpretation, developing training resources and dissemination.	Process of public involvement, with reflection on experiences of public partners and outcomes.	Written notes from verbal feedback, a reflective focus group and written researcher reflections	Unclear ‐ data was collated by two researchers.	Co‐authoring evaluation paper.
Willis et al.	2018	Enhancing inclusion of LGBTQ+ residents in care homes.	8 individuals aged 35‐65 years. Two identified as lesbian, three as gay, one as ‘queer’ (indicating sexual and/or gender fluidity) and one as transgender. Individuals were from White British, Jewish, British‐Asian, and Bangladeshi backgrounds.	To work as community advisors within care homes speaking with residents, staff and other stakeholders, carrying out document reviews and an audit and assessment exercise. Additional input into evaluation study and dissemination of findings.	Process of enabling co‐produced research in long‐term residential care settings for older people.	Semi‐structured interviews. Notes and transcripts from four meetings between public partners and managers were also included as additional data‐sources.	Unclear – resulting reflections from the team were presented.	Co‐authoring evaluation paper.
Worsley et al.	2022	Improving quality of therapeutic relationships between psychiatrists and service users.	11 members of the public identifying as a mental health service user or carer.	Development of a workable research proposal to be submitted to a national funder.	Process of co‐producing a mental health‐related research proposal.	Semi‐structured interviews	Thematic Analysis, inductive approach by an independent researcher	Co‐design of semi‐structured interview, providing reflections/feedback of experience(s), public partner co‐author.

^a^ Includes contribution to data collection activity as participants (e.g. taking part in focus groups or interviews) and involvement activity (e.g. contributing to data analysis/interpretation).

Studies evaluated PI across a broad range of topics and populations, for example, older adults (*n* = 5), parent/carers (*n* = 3), mental health service users (*n* = 2), the LGBTQ+ community (*n* = 3) and those with chronic health conditions (*n* = 3). Public partners were involved in the original (evaluated) studies across the research cycle from initiating project topics to disseminating findings. Data collection and analysis methods were reported inconsistently across included evaluations. For example *n* = 6 studies reported ‘collation’ or ‘synthesis’ of reflective accounts/data, or theme development via meetings, without providing further details of how this was achieved (see Table 1 for full summary).

### 3.3 Data Synthesis

**Figure 3:**
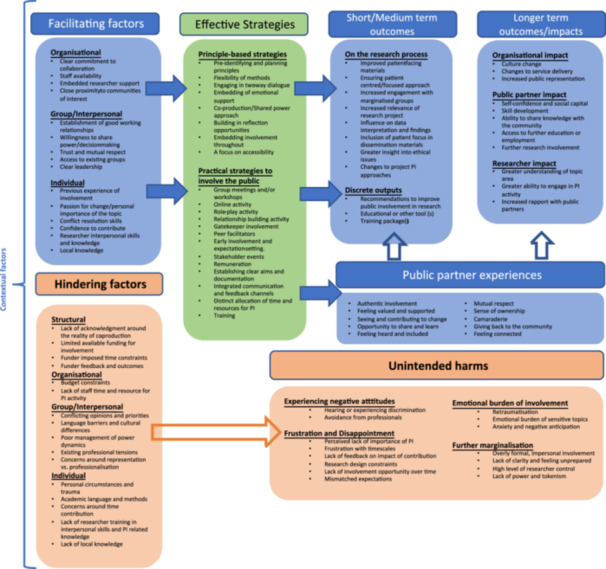
Model of evidence‐based, meaningful public involvement in applied health and social care research, with reference to hindering contextual factors and potential unintended harms.

Main findings are summarised within a model which outlines key components of meaningful PI in applied health and social care research (see Figure 3). This model demonstrates how PI strategies are facilitated by contextual factors at multiple levels, and in turn result in a broad range of outcomes and impacts. The model also includes suggested links (outline arrows) whereby (a) positive public partner experiences facilitate positive outcomes and (b) contextual factors that hinder PI can exacerbate unintended harms for public partners. A narrative summary of findings is included below, with illustrative excerpts included in Table 2. Additional illustrative excerpts are included in Supporting Information G.

**Table 2:** Illustrative quotes from included studies.

Theme	Illustrative Excerpt
**Contextual factors**	
Structural	Despite all the rhetoric around NIHR for public engagement, and you get to write about this on one of the boxes on the forms, I think the system's commitment to an authentic messy public engagement is not there yet. If your public engagement gets you to a pristine application that looks like it was written by a bunch of academics, that's what they want but ours didn't turn out like that (Researcher quote) − Worsley et al. [22]
Organisational	… it was recognized that community partners have other commitments to work and family that take priority and require flexibility. However, the different roles and responsibilities of the academics meant that time available for engagement varied widely, which could, at times, leave some feeling isolated from the process (Author text) – Thomas et al. [25]
Group/Interpersonal	One [public partner group] comprised of adults with intellectual disabilities and had been facilitated by two healthcare professionals…for a number of years. One of the researchers had also worked with the group over this time and had built up a relationship with them, negating the need to build up trust prior to commencing the study (Author text) – Beighton et al. [36] One respondent [was] uncertain whether public members fulfilled all expectations and roles because they had not been challenging enough and believed they were in danger of becoming too integrated within the study team: “Are you becoming professional researchers? Or are you still genuinely spokespeople for public and patients… that's your job, isn't it?” (Researcher quote) – Evans et al. [34]
Individual‐level factors	…the more of this sort of work you do, the better you get it actually challenging researchers, because it takes a lot of confidence to do that, when you start off you're not very confident. (Public partner quote) – Evans et al. [34] I think one of the difficulties is that all the other members of the research team are full‐time researchers and working on the project and it's quite difficult with the [public partners] ‐ you feel quite conscious that you are taking up someone else's time when they could be doing something else. (Researcher quote) – Brett et al. [28]
**Public involvement strategies**	
Principle‐based strategies	…[there's] a lot of support to us to get here for a start and to have good paperwork which we can understand. You know, you don't overwhelm us with stuff, the stuff that we receive is relevant, we can understand it. (Public partner/Dementia patient quote) – Litherland et al. [38]
Practical strategies	Support and training for [PI] activity was delivered in two ways: in locality meetings at each research site and in cross‐site meetings for the five [public partners] across the three sites. This created a working relationship with one researcher at each site, a wider peer support group, as well as fostering relationships with the wider team. (Author text) – Froggatt et al. [29] Social activities, such as going for dinner or drinks outside of more formal research activities, fostered relationships between the research teams and [public partners] and were seen as important to facilitating engagement within the project. (Author text) – Brett et al. [28]
**Outcomes**	
Organisational impact	Academics and health practitioners remarked in their conference evaluation forms that [public partners] level of involvement [in planning/delivering a conference] was an unusual and welcomed element of the event. Listening first‐hand to people's lived experiences challenged their assumptions and increased their understanding of, and receptivity to, key messages around patient's lives, expectations and concerns. A number of attendees commented how these presentations made them reflect on their own practice… (Author text) – Thomas et al. [25]
Changes to the research process	They have been involved in lots of parts … putting the questionnaires together … topic guides for the interviews … general feedback on what was coming out [from the transcripts]. They've had input on papers, meetings, presentations, all that sort of thing. And I think what they've been really good at is driving on the dissemination side of things and making sure the findings make a difference. They've been very active on that and made it clear that that's an expectation from them (Researcher) – Brett et al. [28] I don't think the very elderly people would have disclosed as much to students or young academics, as they were often ashamed of their problems such as fear of computers, severe deafness, using a commode… (Public partner) – Buffel [40]
Impact on individual(s)	Doing this project has enriched me. Kind of immensely and… I wanted to use a different word than confidence… it's just… empowered me. (Public partner/Aphasia patient quote) – McMenamin et al. [39] I have learnt how to be a more sensitive listener. (Public partner quote) – Rowe [35] Meeting and hearing about the experiences of the participants in the survey resulted in considerable reflection and learning for the researchers. Being exposed to many different ways of life had mostly led them to re‐evaluate their own assumptions (Author text) – Rowe [35]
Discrete outputs (of involvement)	We asked [public partners] to design and run a half‐day training course on interviewing people with psychosis for our trial research teams, which was extremely valuable and in hindsight, I wish we'd done it earlier. (Researcher quote) – Slade et al. [37]
**Experiences of public partners**	
Meaningful involvement	I genuinely felt, and I've said this to various people, but this wasn't just a tick box exercise, ooh yes, I've consulted carers, it was a genuine… let's see how you can get involved and I'd like to incorporate your ideas in it, so it did feel like genuine involvement which was great. (Public partner quote) – Beighton et al. [36] Imagine being an outsider to the research world; a Somali mum of a child with autism. Imagine the point at which you realise that you became a researcher, familiar with research processes, ethical considerations, interviewing styles, data analysis and presenting research findings for different audiences. Imagine realising how things can change for you, for your child and for your community. – (Public partner quote), Aabe et al. [32]
Unintended adverse consequences of involvement	Although the research team had some sympathy towards our viewpoint, they chose to focus on the positivist tradition of research as they felt it generated data that was more valid and the research would be more respected and meaningful. There was much discussion and researchers listened at the start, but then focused very much on the approach they wished to take. (Public partner quote) – Slade et al. [37] One thing I didn't anticipate was how much this experience touched on (my) painful memories of homophobia. For me, it was about being mindful of that personal impact. (Public partner quote) – Willis et al. [26] Walking into a room of academics – if you're like us, low‐income, depressed, got issues […] you automatically feel back‐footed as if you don't belong there and they are looking down on you (Public partner quote) – Thomas et al. [25] Is anyone going to pay any attention to it when we get the findings? Will the Local Authority actually pay any attention and do anything about what the people are saying? (Public partner quote) – Buffel et al. [40]

#### 3.3.1 Contextual Factors

##### 3.3.1.1 Structural

Structural barriers, within the present review, referred to the wider funding context research teams worked within, and were often reported as limiting PI, as opposed to other levels that had more variable influence. This related to funder‐imposed time constraints for developing and/or delivering projects [25, 26, 27] as well as a lack of available funding for involving public partners at the grant development stage [27, 28, 29, 30, 31] which, in turn, did not allow for the development of genuine partnership working. These funder‐imposed barriers suggested a lack of acknowledgement for the realities of PI, whereby funders required involvement to take place but then (albeit unintentionally) created barriers to meaningful activity [27].

##### 3.3.1.2 Organisational

Organisational factors were discussed in relation to the research delivery organisations researchers were affiliated with. Studies suggested research organisations could have both a facilitative or hindering effect on PI, particularly in terms of the level of support research staff had to develop and include PI within their work. Research teams felt PI was underpinned by the organisation's overall capacity and willingness to collaborate with public partners [26, 32, 33, 34], ensuring adequate staffing levels and embedded support [25, 27, 28, 29, 30, 32, 34, 35]. The provision of resource and support for those leading PI was felt to be crucial to enabling meaningful involvement opportunities for public partners (i.e. given the resource‐intensive nature of activities).

##### 3.3.1.3 Group/Interpersonal Factors

Facilitating factors at group‐level related to researchers having access to existing public groups or networks [27, 29, 30, 32, 33, 36, 37, 38] established good working relationships, trust and mutual respect [27, 28, 32, 38, 39] and clear leadership of PI [26, 28, 33, 34, 37].

Conflicting opinions and priorities [26, 27, 28, 35, 37], language barriers and cultural differences between public partners and researchers [30, 32] and existing professional tensions (e.g., between researchers and practitioners involved within the research projects) [31] seemed to hinder PI, sometimes leading non‐researchers to question the level of influence public partners genuinely had. Concerns around representation versus professionalisation of public partners also created tensions which hindered partnership working [25, 30, 34].

##### 3.3.1.4 Individual‐Level Factors

Individual‐level factors related to both public partners and researchers. Personal importance for the topic, a passion for change [27, 30, 33, 38], local knowledge of the area/community [28, 40] and conflict resolution skills [27] were mentioned as facilitators of PI. Previous positive experiences of involvement, for both researchers and public partners, also had the potential to facilitate more meaningful involvement opportunities [28, 29, 30, 32, 35, 36, 37, 41] as well as the confidence to contribute in mixed‐group settings [28, 31, 32, 33, 34, 37].

Conversely, difficulties understanding academic language and methods [26, 27, 32, 33, 37], complex personal circumstances and/or past trauma [27, 29, 30], researcher interpersonal skills and PI‐related knowledge [28, 31, 33, 35, 37], and concerns around public partners required time contribution [28, 29, 30, 34] had the potential to hinder PI.

#### 3.3.2 Public Involvement Strategies

Both principle‐based and practical strategies were reported. Multiple strategies, across both categories, were integrated across projects, often tailored to the populations that researchers were working alongside.

##### 3.3.2.1 Principle‐Based Strategies

The majority of studies suggested underpinning principles of involvement that influenced the approach to working in partnership with the public. It was felt there was value in identifying important values and principles and making these explicit from the outset of activity [27, 28, 30, 36, 37, 38, 39]. Key principles centred around the inclusion of flexible methods of involvement [29, 30, 36, 37, 38, 39, 42], engaging in clear two‐way dialogue [28, 30, 33, 38, 39], embedding emotional support for public partners throughout the process [26], aiming for a co‐production/shared power approach [26, 28, 29, 30, 33, 34, 37, 38, 39, 40], ensuring there were opportunities for reflection [29, 30, 33, 40] and a particular focus on increasing accessibility so public partners could participate in team meetings and related activity [29, 33, 38, 41, 42].

##### 3.3.2.2 Practical Strategies

All studies reported a range of strategies to involve public partners. Group/team meetings and workshops, in an accessible format/location, were most commonly reported as a valuable approach that enabled the sharing of ideas, development of projects and facilitated public partner input [26, 27, 28, 29, 30, 31, 33, 34, 35, 36, 37, 38, 39].

Other PI strategies ranged from the involvement of ‘gatekeepers’ or trusted individuals [36, 37, 38, 41], early involvement and management of expectations [28, 29, 30, 33, 34, 39], remuneration for public partner's time and contribution [26, 28, 29, 30, 33, 34, 35, 37, 38, 41], integrated communication and feedback channels [28, 29, 37, 38] and planned relationship‐building activity [26, 27, 28, 37, 38, 39] (see Figure 3 for full list). As indicated above, multiple strategies were included to enhance meaningful opportunities for public partners to collaborate as part of the research team(s).

#### 3.3.3 Outcomes

Outcomes of involvement were not formally measured but anecdotally discussed via interviews, focus groups and reflective sessions within study teams. A summary of reported impacts and outcomes is included below.

##### 3.3.3.1 Organisational Impact

Four studies referred to outcomes at an organisational level including changes to service delivery [26, 37], increased patient/public representation within the organisation [25] and the beginnings of culture change – whereby staff began to see the benefits and/or advocate for PI and reflect on their own practice as a result [25, 26, 34].

##### 3.3.3.2 Changes to the Research Process

Most commonly, authors discussed the impact of PI on the research process and dissemination. For example, PI resulted in more accessible patient‐facing materials [28, 34, 37, 38, 41], ensured a patient‐centred research approach [28, 30, 33, 34, 37, 38, 43] and resulted in greater patient focus in the development of dissemination materials [28, 30, 34, 35, 37, 38, 39, 43].

Increased public partner presence was also reported as beneficial when trying to increase engagement with marginalised communities or individuals, especially when those with lived experience took on peer researcher roles (i.e. conducting research tasks such as recruiting and interviewing participants) [28, 29, 30, 35, 39, 40, 41].

##### 3.3.3.3 Impacts on Individual(S)

Studies reported a range of impacts on both public partners and researchers. For public partners, these impacts included increases in self‐confidence and social capital [25, 27, 28, 35, 36, 38, 39, 40, 41], a greater ability to share knowledge with their community [28, 30, 39], skill development [30, 35, 39, 40] and enabling uptake of further education, employment and/or research activity [33, 35, 40].

Researchers reported personal and professional benefits to working collaboratively with public partners on projects. Engagement in PI facilitated greater insight and understanding into the topic area they were working within [28, 35, 37], and increased their knowledge on working alongside, and building rapport with, communities [28, 30, 37, 41].

##### 3.3.3.4 Discrete Outputs (Of Involvement)

Some of the included studies reported development of educational or other tools such as books, short films, a measurement of recovery support [25, 37, 38] and public partner developed and/or led training for researchers and practitioners [25, 37, 38].

#### 3.3.4 Experiences of Public Partners

##### 3.3.4.1 Meaningful Involvement

Positive experiences highlighted components of meaningful involvement from a public partner perspective and were underpinned by authentic involvement [28, 36], ability to see and contribute to change [26, 30, 32, 36, 37, 39, 40], feeling valued and supported [26, 28, 29, 33, 34, 35, 36, 37, 38, 39, 41] and gaining opportunities to share and learn knowledge [27, 28, 30, 31, 33, 36, 37, 38, 39, 40]. Public partners also discussed positive experiences arising from their increased ability to ‘give back’ or support their community [30, 32, 35, 40].

##### 3.3.4.2 Unintended Adverse Consequences of Involvement

Negative experiences of involvement reflected unintended harms of PI. This related to feelings of frustration and disappointment across a range of different aspects and stages of the involvement process including frustration with timescales, lack of feedback on the impact on public partner's contributions, lack of involvement opportunities over time, mismatched expectations between public partners and researchers and general research design constraints [25, 26, 27, 28, 30, 35, 36, 37, 38, 40, 41].

In addition, public partners recalled being exposed to discriminatory attitudes and/or experienced negative emotional impact of PI activities [25, 26, 29, 35, 36, 39, 40]. For example, some public partners reported hearing offensive and/or discriminatory comments towards LGBTQ+ and ethnically minoritised communities, which had the ability to also trigger past trauma.

Some public partners expressed feelings of further marginalisation [25, 26, 27, 30, 32, 35, 37, 42], particularly when PI activity or materials felt inaccessible to a lay audience.

These adverse consequences of involvement also exacerbated power imbalances whereby public partners questioned their ability to truly influence change beyond the individual research projects they had given their time and experience to [25, 35, 37, 40].

## 4 Discussion

This rapid systematic review synthesised published evaluations of PI in research to gain a deeper understanding of key contextual factors, effective strategies, reported outcomes and public partner experiences (including unintended harms) that underpin meaningful PI. Moreover, our synthesis of public partner experiences demonstrates the nature of *meaningful* involvement; whereby public partners felt genuinely included, valued and supported in research activities, where they have a range of opportunities to contribute to positive change, and also ‘give back’ to their communities via research. In this way, meaningful PI can therefore be linked to improved outcomes and impacts for individuals, the research process and beyond (i.e. as in Figure 3).

These findings are in line with others in the area which highlight the value of ensuring genuine (i.e. rather than tokenistic or ‘tick‐box’) PI approaches, where two‐way learning can take place [44, 45, 46, 47]. We identified several key factors that facilitated PI at the organisational, group/interpersonal and individual levels. These findings support previous research which demonstrates the importance of an organisational culture that actively integrates innovative community engagement and PI into the research infrastructure [44, 48, 49, 50], in addition to the development of equitable, respectful and well‐resourced relationships between researchers and public partners [45, 51]. The present review also identified a range of key characteristics, relevant to all partners, such as strong conflict resolution skills, that can further enhance meaningful PI. Indeed, such factors have previously been found to have a substantial impact on quality of collaboration, inclusivity of the research process, and relevance of the research outcomes [5, 52]. It is of note that structural factors such as funding‐imposed time constraints were highlighted as a barrier to meaningful PI, emphasising the need for more flexible funding mechanisms that allow for early and continuous PI. Our findings support previous calls for greater time allocation before and within grants for meaningful engagement and relationship‐building with public partners [5].

A range of effective strategies were identified and suggest that pre‐identified key principles of involvement and/or co‐production (i.e. where all partners work together in an equal partnership for equal benefit) [53] were an important place to begin. Key components included flexibility, ability for shared‐decision making and accessibility [23, 54, 55, 56]. Multiple practical strategies, tailored to the population of interest, were employed to maximise opportunities for public partners to collaborate with professionals and share their views [46, 57]. However, some key strategies identified within this review, such as remuneration for all PI, early involvement of public partners and PI training for researchers are, to date, not yet routinely implemented across all fields of research [45, 52, 58]. This review suggests that a focus on resolving challenges in implementing these key strategies are likely to increase more authentic forms of partnership working.

Both public partners and researchers reflected on outcomes of PI, most commonly this related to changes to the research process, including development of more ‘patient‐friendly’ approaches, and improved engagement with marginalised groups. These findings are in line with others that show the intrinsic value of PI in enhancing the credibility and acceptability of research processes and findings, i.e. given these are perceived to be grounded in real‐world experiences and contexts, with greater potential for positive change [5, 59]. Longer‐term impacts for organisations, public partners and researchers identified as part of this review also align with those reported across a variety of different settings and contexts [20, 47, 59] and support the view that diverse, high‐quality PI strengthens health and social care research as well as service delivery outcomes [3, 60].

Public partner experience captured within this review identifies important qualities of PI from a lay perspective. Notably, several unintended consequences, or harms, of PI were also identified, such as further marginalisation of public partners, being exposed to discrimination, and multiple causes of frustration [8, 52, 58]. These persistent challenges suggest more structured support and clear communication channels from the outset of PI, as well as broader work to reduce structural‐level barriers to authentic involvement [45]. Given negative or unintended consequences of PI are currently under‐reported in evaluations of PI [2, 47, 61] research organisations should aim to identify and minimise the likelihood of these potential unintended consequences at the earliest possible stages. Without this, there is a risk of worsening existing inequalities and potential loss of innovative insights and contributions [62, 63, 64].

### 4.1 Strengths and Limitations

The present study presents a detailed, evidence‐based review of PI in applied health and social care research. The included model (see Figure 3) provides a holistic view of the complex dynamics affecting PI, including the often under‐reported, negative unintended consequences of PI [2]. This model gives research funders, organisations and individuals key areas for prioritisation to reduce tokenism and support the ongoing development of genuine and meaningful PI. The rigorous methods and quality assessment applied to this review have also outlined methodological improvements to advance evaluation of PI within the area. Importantly, the inclusion of public partners within our core study team shaped our understanding and focus (particularly regarding the inclusion of unintended harms) and increased authenticity of findings, grounding these in lived experience. This approach has also facilitated the identification of relevant future priorities, as outlined below.

There are also limitations to consider. PI in health and social care research (and the evaluation of this) is often described using a broad range of terms and phrases. For example, ‘public involvement’ has been referred to as ‘patient involvement’, ‘co‐production’, ‘co‐design’ and ‘consumer involvement’ amongst other terms. As such, it is possible that eligible studies may have been missed during the review process. However, we worked closely with an Information Specialist to develop our search strategy; supplementing this with additional hand‐searching of key journals in the area to reduce the likelihood of missing key papers. Strict inclusion/exclusion criteria were also applied in light of our review scope and context; this included the exclusion of studies conducted outside of the UK as well as grey literature that may have included useful findings. Given our findings align with broader PI literature, including those from other fields such as clinical trial research [61, 65, 66], we suggest that the results reported here may still be applicable to other regions and contexts. Future research may wish to apply fewer exclusion criteria for a broader review, however, this should be balanced with careful consideration of context, and the impact this has on findings [46]. Given the limited number of evaluations, which often focused on group and individual level factors, there may also be additional components to meaningful PI that are not reported in our model. With further improvement and development of evaluation approaches it is recommended that this model is therefore updated in future to reflect progression in the field. Finally, the need to develop and deliver accessible training and support throughout the project added additional time to the review process. This suggests a need for more widely available, accessible training in review methodologies, to facilitate diverse PI in literature reviews.

### 4.2 Recommendations for Future Research

We observed variable reporting quality from both a methodological and PI standpoint during the process of our review as well as a lack of outcome evaluations; similar findings have been observed in patient safety research and health service design [67, 68]. This introduces challenges when attempting to fully understand the process, outcomes and impacts of PI within the field. As such future research should focus on developing more robust, transparent and appropriate evaluation approaches to enhance our understanding of meaningful, inclusive PI in applied health and social care research. This should include adherence to standardised reporting guidelines [12, 67], a focus on what is being measured and why [8, 46, 69] and consideration of independent and/or community‐led evaluation of PI activities [63]. These approaches could mitigate potential biases and ensure that the perspectives of public partners are accurately represented. Importantly, independent evaluations may facilitate the reporting of unintended negative consequences of PI, enhance transparency of PI activities and help foster greater trust and engagement among all stakeholders.

Efforts should also be made to include a broader range of public partners in PI activities, particularly those with minimal knowledge or experience of research. Whilst it is of clear value to include experienced public partners, particular effort should focus on ensuring diverse representation of experiences so aforementioned benefits of PI are more equitably distributed. Future research should therefore develop inclusive PI strategies that address barriers to participation and promote diversity for example through partnerships with community organisations or targeted outreach programmes [70, 71].

Additionally, our synthesis resulted in an evidence‐based model of meaningful PI. Facilitation is required at different levels to ensure genuine and sustainable partnership working and to minimise unintended harms. Funders have a prominent role in minimising structural barriers and supporting more diverse forms of PI. Our model can serve as a foundation to be tailored to more specific contexts, for example to support meaningful partnerships within a range of underserved communities for example ethnically minoritised communities, those with learning disabilities, those living in areas of high deprivation and so on.

## 5 Conclusion

This review highlights key contextual factors, strategies, outcomes and public partner experiences that underpin ‘meaningful’ PI in applied health and social care research. Our findings outline the need for PI to be facilitated at multiple levels, with targeted focus on reducing unintended harms and further marginalisation of traditionally excluded groups. We support the view that ‘one size fits all’ methodology is not appropriate nor conducive to diverse PI in health and/or social care research and suggest that tailored, principle‐based approaches, in conjunction with practical support from research delivery organisations and funders can enable more impactful involvement to take place. More transparent and robust approaches to evaluating PI, co‐produced with public partners, are also critical to further knowledge in this area. The included model provides an accessible summary of priority areas for researchers, organisations and funders going forward, and a foundation to support evidence‐based planning, delivery and evaluation of high‐quality PI in health and social care research.

## Author Contributions

**Angela Wearn:** conceptualisation, methodology, writing–original draft, project administration, formal analysis, investigation, visualisation. **Kerry Brennan‐Tovey:** methodology, formal analysis, writing–review and editing, investigation, visualisation. **Emma A. Adams:** methodology, writing–review and editing, investigation, visualisation. **Hayley Alderson:** methodology, writing–review and editing, investigation, visualisation. **Judy Baariu:** writing–original draft, writing–review and editing, visualisation. **Mandy Cheetham:** methodology, writing–review and editing, investigation. **Victoria Bartle:** methodology, investigation, formal analysis, writing–review and editing, visualisation. **Lucy Palfreyman:** methodology, writing–review and editing, formal analysis, investigation, visualisation. **Violet Rook:** methodology, writing–review and editing, formal analysis, investigation, visualisation. **Felicity Shenton:** methodology, writing–review and editing. **Sheena E. Ramsay:** conceptualisation, methodology, writing–review and editing, visualisation. **Eileen Kaner:** conceptualisation, methodology, writing–review and editing, funding acquisition, visualisation.

## Conflicts of Interest

Eileen Kaner is the Director of the NIHR Applied Research Collaboration North East and North Cumbria. All other authors declare no conflicts of interest.

## Supporting information

Supporting information.

## Data Availability

The authors have nothing to report.

## References

[1] S. R. Arnstein , “A Ladder of Citizen Participation,” Journal of the American Institute of Planners 35, no. 4 (1969): 216–224, 10.1080/01944366908977225.

[2] J. Russell , T. Greenhalgh , and M. Taylor . Patient and Public Involvement in NIHR Research 2006–2019: Policy Intentions, Progress and Themes. 2019, https://oxfordbrc.nihr.ac.uk/wp-content/uploads/2019/05/NIHR-and-PPI-report-Feb_2019.pdf.

[3] NIHR . Best Research for Best Health: The Next Chapter. 2021, https://www.nihr.ac.uk/reports/best-research-for-best-health-the-next-chapter/34535#our-areas-of-strategic-focus.

[4] NIHR . Shared Commitment to Public Involvement. 2022, https://www.nihr.ac.uk/documents/shared-commitment-to-public-involvement/30134.

[5] J. Brett , S. Staniszewska , C. Mockford , et al., “Mapping the Impact of Patient and Public Involvement on Health and Social Care Research: A Systematic Review,” Health Expectations 17, no. 5 (2014): 637–650, 10.1111/j.1369-7625.2012.00795.x. 22809132 PMC5060910

[6] J. Brett , S. Staniszewska , C. Mockford , et al., “A Systematic Review of the Impact of Patient and Public Involvement on Service Users, Researchers and Communities,” Patient–Patient‐Centered Outcomes Research 7, no. 4 (2014): 387–395, 10.1007/s40271-014-0065-0.pdf. 25034612

[7] K. Staley , S. A. Buckland , H. Hayes , M. Tarpey , et al., “‘The Missing Links’: Understanding How Context and Mechanism Influence the Impact of Public Involvement in Research,” Health Expectations 17, no. 6 (2014): 755–764, 10.1111/hex.12017. 23107054 PMC5060928

[8] J. Russell , N. Fudge , and T. Greenhalgh , “The Impact of Public Involvement in Health Research: What Are We Measuring? Why Are We Measuring It? Should We Stop Measuring It?,” Research Involvement and Engagement 6, no. 1 (2020): 63. 33133636 10.1186/s40900-020-00239-w PMC7592364

[9] M. J. Page , J. E. McKenzie , P. M. Bossuyt , et al., “The PRISMA 2020 Statement: An Updated Guideline for Reporting Systematic Reviews,” BMJ 372 (2021): n71, 10.1136/bmj.n71. 33782057 PMC8005924

[10] C. Garritty , G. Gartlehner , B. Nussbaumer‐Streit , et al., “Cochrane Rapid Reviews Methods Group Offers Evidence‐Informed Guidance to Conduct Rapid Reviews,” Journal of Clinical Epidemiology 130 (2021): 13–22, 10.1016/j.jclinepi.2020.10.007. 33068715 PMC7557165

[11] A. Tong , K. Flemming , E. McInnes , S. Oliver , and J. Craig , “Enhancing Transparency in Reporting the Synthesis of Qualitative Research: ENTREQ,” BMC Medical Research Methodology 12 (2012): 181. 23185978 10.1186/1471-2288-12-181 PMC3552766

[12] S. Staniszewska , J. Brett , I. Simera , et al., “GRIPP2 Reporting Checklists: Tools to Improve Reporting of Patient and Public Involvement in Research,” BMJ 358 (2017): 3453, https://www.bmj.com/content/358/bmj.j3453. 10.1136/bmj.j3453 PMC5539518 28768629

[13] Critical Appraisal Skills Programme . CASP Qualitative Studies Checklist. 2023, https://casp-uk.net/casp-tools-checklists/qualitative-studies-checklist/.

[14] J. Dalton , A. Booth , J. Noyes , and A. J. Sowden , “Potential Value of Systematic Reviews of Qualitative Evidence in Informing User‐Centered Health and Social Care: Findings From a Descriptive Overview,” Journal of Clinical Epidemiology 88 (2017): 37–46, 10.1016/j.jclinepi.2017.04.020. 28450254

[15] H. A. Long , D. P. French , and J. M. Brooks , “Optimising the Value of the Critical Appraisal Skills Programme (CASP) Tool for Quality Appraisal in Qualitative Evidence Synthesis,” Research Methods in Medicine & Health Sciences 1, no. 1 (2020): 31–42, 10.1177/2632084320947559.

[16] R. Garside , “Should We Appraise the Quality of Qualitative Research Reports for Systematic Reviews, and If so, How?,” Innovation: The European Journal of Social Science Research 27, no. 1 (2014): 67–79, 10.1080/13511610.2013.777270.

[17] M. Sandelowski , S. Docherty , and C. Emden , “Qualitative Metasynthesis: Issues and Techniques,” Research in Nursing & Health 20, no. 4 (1997): 365–371. 9256882 10.1002/(sici)1098-240x(199708)20:4<365::aid-nur9>3.0.co;2-e

[18] J. Ritchie , J. Lewis , C. McNaughton Nicholls , and R. Ormston , Qualitative Research Practice: a Guide for Social Science Students and Researchers (London: Sage, 2014).

[19] J. Thomas , A. O'Mara‐Eves , A. Harden , and M. Newman , “Synthesis Methods for Textual or Mixed Methods Data,” An Introduction to Systematic Reviews (2017): 181–210, http://discovery.ucl.ac.uk/1551097/.

[20] Y. Bombard , G. R. Baker , E. Orlando , et al., “Engaging Patients to Improve Quality of Care: A Systematic Review,” Implementation Science 13, no. 1 (2018): 98. 30045735 10.1186/s13012-018-0784-z PMC6060529

[21] K. Beckett , M. Farr , A. Kothari , L. Wye , and A. Le May , “Embracing Complexity and Uncertainty to Create Impact: Exploring the Processes and Transformative Potential of Co‐Produced Research Through Development of a Social Impact Model,” Health Research Policy and Systems 16, no. 1 (2018): 118. 30537975 10.1186/s12961-018-0375-0 PMC6288891

[22] C. Bonell , F. Jamal , G. J. Melendez‐Torres , and S. Cummins , “‘Dark Logic’: Theorising the Harmful Consequences of Public Health Interventions,” Journal of Epidemiology and Community Health 69, no. 1 (2015): 95–98, https://pubmed.ncbi.nlm.nih.gov/25403381/. 25403381 10.1136/jech-2014-204671

[23] NIHR . UK Standards for Public Involvement, (2019), https://drive.google.com/file/d/1U-IJNJCfFepaAOruEhzz1TdLvAcHTt2Q/view.

[24] NIHR Centre for Engagement and Dissemination . Payment Guidance for Researchers and Professionals. (2021).

[25] F. Thomas , L. Hansford , K. Wyatt , et al., “An Engaged Approach to Exploring Issues Around Poverty and Mental Health: A Reflective Evaluation of the Research Process From Researchers and Community Partners Involved in the Destress Study,” Health Expectations 24, no. S1 (2021): 113–121. 32449304 10.1111/hex.13065 PMC8137483

[26] P. Willis , K. Almack , T. Hafford‐Letchfield , P. Simpson , B. Billings , and N. Mall , “Turning the Co‐Production Corner: Methodological Reflections From an Action Research Project to Promote LGBT Inclusion in Care Homes for Older People,” International Journal of Environmental Research and Public Health 15, no. 4 (2018): 695. 29642460 10.3390/ijerph15040695 PMC5923737

[27] J. D. Worsley , M. McKeown , T. Wilson , and R. Corcoran , “A Qualitative Evaluation of Coproduction of Research: ‘If You Do It Properly, You Will Get Turbulence’,” Health Expectations 25, no. 5 (2022): 2034–2042. 33949751 10.1111/hex.13261 PMC9615072

[28] J. Brett , Z. Davey , F. Matley , et al., “Impact of Patient and Public (PPI) Involvement in the Life After Prostate Cancer Diagnosis (LAPCD) Study: A Mixed‐Methods Study,” BMJ Open 12, no. 11 (2022): e060861. 10.1136/bmjopen-2022-060861 PMC9664269 36375983

[29] K. Froggatt , C. Goodman , H. Morbey , et al., “Public Involvement in Research Within Care Homes: Benefits and Challenges in the APPROACH Study,” Health Expectations 19, no. 6 (2016): 1336–1345, 10.1111/hex.12431. 26620796 PMC5139055

[30] N. Cotterell and T. Buffel , “‘Holders of Knowledge Are Communities, Not Academic Institutions’: Lessons From Involving Minoritised Older People as Co‐Researchers in a Study of Loneliness in Later Life,” Qualitative Research in Psychology 20, no. 3 (2023): 441–470, 10.1080/14780887.2023.2180463.

[31] T. J. Devonport , W. Nicholls , L. H. Johnston , R. Gutteridge , and A. Watt , “It's Not Just ‘What’ You Do, It's Also the ‘Way’ That You Do It: Patient and Public Involvement in the Development of Health Research,” International Journal for Quality in Health Care 30, no. 2 (2018): 152–156. 29346582 10.1093/intqhc/mzx177

[32] N. O. Aabe , F. Fox , D. Rai , and S. Redwood , “Inside, Outside and In‐Between: The Process and Impact of Co‐Producing Knowledge About Autism in a UK Somali Community,” Health Expectations 22, no. 4 (2019): 752–760, 10.1111/hex.12939. 31318129 PMC6737832

[33] L. Forbat , A. Macgregor , T. Brown , et al., “Negotiating Pace, Focus and Identities: Patient/Public Involvement/Engagement in a Palliative Care Study,” Sociology of Health and Illness 46, (2024): 1–18. 38720523 10.1111/1467-9566.13785

[34] B. A. Evans , A. Carson‐Stevens , A. Cooper , et al., “Implementing Public Involvement Throughout the Research Process—Experience and Learning From the GPs in EDs Study,” Health Expectations 25, no. 5 (2022): 2471–2484. 35894169 10.1111/hex.13566 PMC9615054

[35] A. Rowe , “The Effect of Involvement in Participatory Research on Parent Researchers in a Sure Start Programme,” Health and Social Care in the Community 14, no. 6 (2006): 465–473. 17059488 10.1111/j.1365-2524.2006.00632.x

[36] C. Beighton , C. Victor , I. M. Carey , et al., “‘I'm Sure We Made It a Better Study’: Experiences of Adults With Intellectual Disabilities and Parent Carers of Patient and Public Involvement in a Health Research Study,” Journal of Intellectual Disabilities 23, no. 1 (2019): 78–96. 28812949 10.1177/1744629517723485 PMC6383106

[37] M. Slade , P. Trivedi , R. Chandler , and M. Leamy , “Developing Involvement During a Programme of Recovery Research,” Journal of Mental Health Training, Education and Practice 11, no. 4 (2016): 244–255, https://core.ac.uk/download/pdf/196255896.pdf.

[38] R. Litherland , J. Burton , M. Cheeseman , et al., “Reflections on PPI From the ‘Action on Living Well: Asking You’ Advisory Network of People With Dementia and Carers as Part of the IDEAL Study,” Dementia 17, no. 8 (2018): 1035–1044, 10.1177/1471301218789309. 30373457

[39] R. McMenamin , M. Griffin , B. Grzybowska , and C. Pound , “Working Together: Experiences of People With Aphasia as Co‐Researchers in Participatory Health Research Studies,” Aphasiology (2021): 1–22, 10.1080/02687038.2021.1923948.35002009

[40] T. Buffel , “Older Coresearchers Exploring Age‐Friendly Communities: An ‘Insider’ Perspective on the Benefits and Challenges of Peer‐Research,” Gerontologist 59, no. 3 (2019): 538–548. 29401222 10.1093/geront/gnx216

[41] F. E. Lithander , E. Tenison , D. A. Jones , et al., “Working With Public Contributors in Parkinson's Research: What Were the Changes, Benefits and Learnings? A Critical Reflection From the Researcher and Public Contributor Perspective,” Health Expectations 27, no. 1 (2023): e13914, 10.1111/hex.13914. 37990485 PMC10768872

[42] R. Stocker , K. Brittain , K. Spilsbury , and B. Hanratty , “Patient and Public Involvement in Care Home Research: Reflections on the How and Why of Involving Patient and Public Involvement Partners in Qualitative Data Analysis and Interpretation,” Health Expectations 24, no. 4 (2021): 1349–1356. 33974718 10.1111/hex.13269 PMC8369083

[43] J. Sutton and M. Weiss , “Involving Patients as Advisors in Pharmacy Practice Research: What Are the Benefits?,” International Journal of Pharmacy Practice 16, no. 4 (2010): 231–238.

[44] T. Jackson , H. Pinnock , S. M. Liew , et al., “Patient and Public Involvement in Research: From Tokenistic Box Ticking to Valued Team Members,” BMC Medicine 18, no. 1 (2020): 79. 32279658 10.1186/s12916-020-01544-7 PMC7153227

[45] H. Smith , L. Budworth , C. Grindey , et al., “Co‐Production Practice and Future Research Priorities in United Kingdom‐Funded Applied Health Research: A Scoping Review,” Health Research Policy and Systems 20, no. 1 (2022): 36, 10.1186/s12961-022-00838-x. 35366898 PMC8976994

[46] K. Staley and D. Barron , “Learning as an Outcome of Involvement in Research: What Are the Implications for Practice, Reporting and Evaluation?,” Research Involvement and Engagement 5, no. 1 (2019): 14. 30915234 10.1186/s40900-019-0147-1 PMC6416961

[47] A. W. Karlsson , A. Kragh‐Sørensen , K. Børgesen , et al., “Roles, Outcomes, and Enablers Within Research Partnerships: A Rapid Review of the Literature on Patient and Public Involvement and Engagement in Health Research,” Research Involvement and Engagement 9, no. 1 (2023): 43, 10.1186/s40900-023-00448-z. 37322525 PMC10268359

[48] C. Mockford , M. Murray , K. Seers , et al., “A SHARED Study – The Benefits and Costs of Setting up a Health Research Study Involving Lay Co‐Researchers and How We Overcame the Challenges,” Research Involvement and Engagement 2, no. 1 (2016): 1–12, 10.1186/s40900-016-0021-3. 29062509 PMC5611649

[49] K. Cowan . INVOLVE: A Practical Guide to Being Inclusive in Public Involvement in Health Research‐Lessons From the Reaching Out Programme. Southampton, UK; 2020, https://www.invo.org.uk/wp-content/uploads/2019/02/Being-Inclusive-Health‐.

[50] C. A. Marston , R. Matthews , A. Renedo , and J. E. Reed , “Working Together to Co‐Produce Better Health: The Experience of the Collaboration for Leadership in Applied Health Research and Care for Northwest London,” Journal of Health Services Research & Policy 26, no. 1 (2021): 28–36. 32486987 10.1177/1355819620928368 PMC7734957

[51] S. B. Cashman , S. Adeky , A. J. Allen , et al., “The Power and the Promise: Working With Communities to Analyze Data, Interpret Findings, and Get to Outcomes,” American Journal of Public Health 98, no. 8 (2008): 1407–1417. 18556617 10.2105/AJPH.2007.113571 PMC2446454

[52] J. Ocloo , S. Garfield , B. D. Franklin , and S. Dawson , “Exploring the Theory, Barriers and Enablers for Patient and Public Involvement Across Health, Social Care and Patient Safety: A Systematic Review of Reviews,” Health Research Policy and Systems 19, no. 1 (2021): 8, 10.1186/s12961-020-00644-3. 33472647 PMC7816359

[53] Co‐Production Collective . What Does Co‐production Mean to Us? 2021, https://www.coproductioncollective.co.uk/what-is-co-production/our-approach.

[54] K. Seddon , J. Elliott , M. Johnson , et al., “Using the United Kingdom Standards for Public Involvement to Evaluate the Impact of Public Involvement in a Multinational Clinical Study,” Research Involvement and Engagement 7, no. 1 (2021): 22, 10.1186/s40900-021-00264-3. 33931134 PMC8088001

[55] S. Dawson , A. Ruddock , V. Parmar , et al., “Patient and Public Involvement in Doctoral Research: Reflections and Experiences of the PPI Contributors and Researcher,” Research Involvement and Engagement 6, no. 1 (2020): 23. 32426162 10.1186/s40900-020-00201-w PMC7216324

[56] A. Albert , S. Islam , M. Haklay , and R. R. C. McEachan , “Nothing About Us Without Us: A Co‐Production Strategy for Communities, Researchers and Stakeholders to Identify Ways of Improving Health and Reducing Inequalities,” Health Expectations 26 (2023): 836–846. 36683204 10.1111/hex.13709 PMC10010091

[57] N. Fudge , C. D. A. Wolfe , and C. McKevitt , “Assessing the Promise of User Involvement in Health Service Development: Ethnographic Study,” BMJ 336, no. 7639 (2008): 313–317. 18230646 10.1136/bmj.39456.552257.BE PMC2234509

[58] E. Agyei‐Manu , N. Atkins , B. Lee , et al., “The Benefits, Challenges, and Best Practice for Patient and Public Involvement in Evidence Synthesis: A Systematic Review and Thematic Synthesis,” Health Expectations 26, no. 4 (2023): 1436–1452. 37260191 10.1111/hex.13787 PMC10349234

[59] C. Mockford , S. Staniszewska , F. Griffiths , and S. Herron‐Marx , “The Impact of Patient and Public Involvement on UK NHS Health Care: A Systematic Review,” International Journal for Quality in Health Care 24, no. 1 (2012): 28–38. 22109631 10.1093/intqhc/mzr066

[60] NHS Providers . Co‐production and Engagement With Communities as a Solution to Reducing Health Inequalities. 2024, https://nhsproviders.org/media/698572/co-production-health-ineq-1e.pdf.

[61] R. J. Bergin , C. E. Short , N. Davis , et al., “The Nature and Impact of Patient and Public Involvement in Cancer Prevention, Screening and Early Detection Research: A Systematic Review,” Preventive Medicine 167 (2023): 107412. 36592674 10.1016/j.ypmed.2022.107412

[62] K. A. Gray‐Burrows , T. A. Willis , R. Foy , et al., “Role of Patient and Public Involvement in Implementation Research: A Consensus Study,” BMJ Quality & Safety 27, no. 10 (2018): 858–864. 10.1136/bmjqs-2017-006954 PMC6166593 29666310

[63] S. Blackburn , R. Hine , S. Fairbanks , et al., “The INSIGHT Project: Reflections on the Co‐Production of a Quality Recognition Programme to Showcase Excellence in Public Involvement in Health and Care Research,” Research Involvement and Engagement 9, no. 1 (2023): 99. 37880805 10.1186/s40900-023-00508-4 PMC10601214

[64] United Kingdom Research Institute , Research and Innovation for All: UKRI's Public Engagement Strategy, 2019, https://ukri.org/publications/ukri-public-engagement-strategy/research-and-innovation-for-all-ukris-public-engagement-strategy/.

[65] J. Thompson , P. Bissell , C. L. Cooper , C. J. Armitage , and R. Barber , “Exploring the Impact of Patient and Public Involvement in a Cancer Research Setting,” Qualitative Health Research 24, no. 1 (2014): 46–54. 24277776 10.1177/1049732313514482 PMC4509885

[66] P. Bissell , J. Thompson , and B. Gibson , “Exploring Difference or Just Watching the Experts at Work? Interrogating Patient and Public Involvement (PPI) in a Cancer Research Setting Using the Work of Jurgen Habermas,” Sociology 52, no. 6 (2018): 1200–1216.

[67] S. Hammoud , L. Alsabek , L. Rogers , and E. McAuliffe , “Systematic Review on the Frequency and Quality of Reporting Patient and Public Involvement in Patient Safety Research,” BMC Health Services Research 24, no. 1 (2024): 532, 10.1186/s12913-024-11021-z. 38671476 PMC11046929

[68] N. Lloyd , A. Kenny , and N. Hyett , “Evaluating Health Service Outcomes of Public Involvement in Health Service Design in High‐Income Countries: A Systematic Review,” BMC Health Services Research 21, no. 1 (2021): 364. 33879149 10.1186/s12913-021-06319-1 PMC8056601

[69] C. Marston and A. Renedo , “Understanding and Measuring the Effects of Patient and Public Involvement: An Ethnographic Study.” Lancet. 382, no. S69 (2013), 10.1016/S0140-6736(13)62494-0.

[70] S. Hatch , J. Fitzgibbon , A. J. Tonks , and L. Forty , “Diversity in Patient and Public Involvement in Healthcare Research and Education—Realising the Potential,” Health Expectations 27, no. 1 (2024): e13896, 10.1111/hex.13896. 37867364 PMC10726264

[71] S. Islam , O. Joseph , A. Chaudry , et al., “‘We Are Not Hard to Reach, but We May Find It Hard to Trust’. Involving and Engaging ‘Seldom Listened To’ Community Voices in Clinical Translational Health Research: A Social Innovation Approach,” Research Involvement and Engagement 7, no. 1 (2021): 46. 34174961 10.1186/s40900-021-00292-z PMC8234650

