# Supplementary material for: Evaluating Process and Outcomes of Public Involvement in Applied Health and Social Care Research: A Rapid Systematic Review
Source: Health Expect. 2025 Jan 22;28(1):e70160. doi: 10.1111/hex.70160 (PMC11751718; doi:10.1111/hex.70160)
Supplement: Supplementary file 1 — Supporting information. [file HEX-28-e70160-s001.docx]

# Reporting checklists

Table A1. ENTREQ checklist

| Item No. | Guide and Description | Report on page no. |
| --- | --- | --- |
| 1. Aim | State the research question the synthesis addresses | 3 |
| 2. Synthesis methodology | Identify the synthesis methodology or theoretical framework which underpins the synthesis, and describe the rationale for choice of methodology (e.g. meta-ethnography, thematic synthesis, critical interpretive synthesis, grounded theory synthesis, realist synthesis, meta-aggregation, meta-study, framework synthesis) | 5 |
| 3. Approach to searching | Indicate whether the search was pre-planned (comprehensive search strategies to seek all available studies) or iterative (to seek all available concepts until they theoretical saturation is achieved) | 3 |
| 4. Inclusion criteria | Specify the inclusion/exclusion criteria (e.g. in terms of population, language, year limits, type of publication, study type) | 4 |
| 5. Data sources | Describe the information sources used (e.g. electronic databases (MEDLINE, EMBASE, CINAHL, psycINFO), grey literature databases (digital thesis, policy reports), relevant organisational websites, experts, information specialists, generic web searches (Google Scholar) hand searching, reference lists) and when the searches conducted; provide the rationale for using the data sources | 3 |
| 6. Electronic Search strategy | Describe the literature search (e.g. provide electronic search strategies with population terms, clinical or health topic terms, experiential or social phenomena related terms, filters for qualitative research, and search limits) | Supp. Materials B |
| 7. Study screening methods | Describe the process of study screening and sifting (e.g. title, abstract and full text review, number of independent reviewers who screened studies) | 4 and Figure 2 |
| 8. Study characteristics | Present the characteristics of the included studies (e.g. year of publication, country, population, number of participants, data collection, methodology, analysis, research questions) | 6-7 and Table 1 |
| 9. Study selection results | Identify the number of studies screened and provide reasons for study exclusion (e.g. for comprehensive searching, provide numbers of studies screened and reasons for exclusion indicated in a figure/flowchart; for iterative searching describe reasons for study exclusion and inclusion based on modifications to the research question and/or contribution to theory development) | Figure 2 - PRISMA flowchart |
| 10. Rationale for appraisal | Describe the rationale and approach used to appraise the included studies or selected findings (e.g. assessment of conduct (validity and robustness), assessment of reporting (transparency), assessment of content and utility of the findings) | 4-5 |
| 11. Appraisal items | State the tools, frameworks and criteria used to appraise the studies or selected findings (e.g. Existing tools: CASP, QARI, COREQ, Mays and Pope [25]; reviewer developed tools; describe the domains assessed: research team, study design, data analysis and interpretations, reporting) | 4-5 and Supp. Materials E |
| 12. Appraisal process | Indicate whether the appraisal was conducted independently by more than one reviewer and if consensus was required | 4 |
| 13. Appraisal results | Present results of the quality assessment and indicate which articles, if any, were weighted/excluded based on the assessment and give the rationale | 6 and Supp. Materials E |
| 14. Data extraction | Indicate which sections of the primary studies were analysed and how were the data extracted from the primary studies? (e.g. all text under the headings “results /conclusions” were extracted electronically and entered into a computer software) | 4 |
| 15. Software | State the computer software used, if any | 4 |
| 16. Number of reviewers | Identify who was involved in coding and analysis | 5 |
| 17. Coding | Describe the process for coding of data (e.g. line by line coding to search for concepts) | 5 |
| 18. Study comparison | Describe how were comparisons made within and across studies (e.g. subsequent studies were coded into pre-existing concepts, and new concepts were created when deemed necessary) | 5 |
| 19. Derivation of themes | Explain whether the process of deriving the themes or constructs was inductive or deductive | 5 |
| 20. Quotations | Provide quotations from the primary studies to illustrate themes/constructs, and identify whether the quotations were participant quotations of the author’s interpretation | Table 2 and Supp. Materials F |
| 21. Synthesis output | Present rich, compelling and useful results that go beyond a summary of the primary studies (e.g. new interpretation, models of evidence, conceptual models, analytical framework, development of a new theory or construct) | 7-11 and Figure 3 |

Table A2. GRIPP2 short-form reporting checklist (from Staniszewska et al., 2017).

| **Section and topic** | **Item** | **Reported on page No** |
| --- | --- | --- |
| 1: Aim | Report the aim of PPI in the study | 5 |
| 2: Methods | Provide a clear description of the methods used for PPI in the study | 5-6 |
| 3: Study results | Outcomes—Report the results of PPI in the study, including both positive and negative outcomes | 7-11 |
| 4: Discussion and conclusions | Outcomes—Comment on the extent to which PPI influenced the study overall. Describe positive and negative effects | 13 |
| 5: Reflections/critical perspective | Comment critically on the study, reflecting on the things that went well and those that did not, so others can learn from this experience | 13 |

# Example Search strategy

OVID EMBASE

1. (((public or citizen* or patient* or carer* or service user* or lay or family or relative* or stakeholder* or community or resident*) adj2 (engag* or advis* or consult* or collaborat* or rights or co-production or co-design)) or ((public or citizen* or patient or carer* or service user* or lay or family or relative* or stakeholder* or community or resident*) adj2 (involvement or participation)) or "expert* by experience" or patient panel*).ti,ab.

2. (((patient or public or consumer or user) adj3 (involvement or participation or advoca$ or engag$ or empower$ or input$ or voice$)) or ((patients or consumers or users) adj3 (advoca$ or engag$ or empower$ or input$ or voice$)) or ((patient or public or consumer or user) adj1 (centr* or center* or focus* or driven)) or patient partner$ or patient research partner$ or "patient$ and famil$ partner$" or (patient$ adj3 partnership$) or (coproduc$ or co-produc$ or coauthor$ or co-author$ or codesign$ or co-design$ or coresearch$ or co-research$) or engaged research or patient participants or participatory.ti.ab.

3. patient advocacy/ or patient empowerment/ or stakeholder engagement/ or patient participation/ or patient engagement/ or consumer/ or citizen science/ or community participation/ or participatory research/

4. or/1-3

5. (research adj2 (process$ or methodol$)).ti,ab.

6. ((involvement or participatory or participation) and (evaluat* or critiqu* or reflect* or impact* or research or design* or co-design* or rationale* or process$ or methodol$)).ti.

7. ((involvement or participatory or participation or PPI or PPIE or "patient and public involvement") adj4 (investigat* or evaluat* or critiqu* or reflect* or impact* or implement* or outcome* or implication* or process$ or methodol$)).ti,ab.

8. or/5-7

9. 4 and 8

10. limit 9 to (english language and yr="2006 -Current")

11. exp United Kingdom/

12. (national health service* or nhs*).ti,ab,in,ad.

13. (english not ((published or publication* or translat* or written or language* or speak* or literature or citation*) adj5 english)).ti,ab.

14. (gb or "g.b." or britain* or (british* not "british columbia") or uk or "u.k." or united kingdom* or (england* not "new england") or northern ireland* or northern irish* or scotland* or scottish* or ((wales or "south wales") not "new south wales") or welsh*).ti,ab,jx,in,ad.

15. (bath or "bath's" or ((birmingham not alabama*) or ("birmingham's" not alabama*) or bradford or "bradford's" or brighton or "brighton's" or bristol or "bristol's" or carlisle* or "carlisle's" or (cambridge not (massachusetts* or boston* or harvard*)) or ("cambridge's" not (massachusetts* or boston* or harvard*)) or (canterbury not zealand*) or ("canterbury's" not zealand*) or chelmsford or "chelmsford's" or chester or "chester's" or chichester or "chichester's" or coventry or "coventry's" or derby or "derby's" or (durham not (carolina* or nc)) or ("durham's" not (carolina* or nc)) or ely or "ely's" or exeter or "exeter's" or gloucester or "gloucester's" or hereford or "hereford's" or hull or "hull's" or lancaster or "lancaster's" or leeds* or leicester or "leicester's" or (lincoln not nebraska*) or ("lincoln's" not nebraska*) or (liverpool not (new south wales* or nsw)) or ("liverpool's" not (new south wales* or nsw)) or ((london not (ontario* or ont or toronto*)) or ("london's" not (ontario* or ont or toronto*)) or manchester or "manchester's" or (newcastle not (new south wales* or nsw)) or ("newcastle's" not (new south wales* or nsw)) or norwich or "norwich's" or nottingham or "nottingham's" or oxford or "oxford's" or peterborough or "peterborough's" or plymouth or "plymouth's" or portsmouth or "portsmouth's" or preston or "preston's" or ripon or "ripon's" or salford or "salford's" or salisbury or "salisbury's" or sheffield or "sheffield's" or southampton or "southampton's" or st albans or stoke or "stoke's" or sunderland or "sunderland's" or truro or "truro's" or wakefield or "wakefield's" or wells or westminster or "westminster's" or winchester or "winchester's" or wolverhampton or "wolverhampton's" or (worcester not (massachusetts* or boston* or harvard*)) or ("worcester's" not (massachusetts* or boston* or harvard*)) or (york not ("new york*" or ny or ontario* or ont or toronto*)) or ("york's" not ("new york*" or ny or ontario* or ont or toronto*))))).ti,ab,in,ad.

16. (bangor or "bangor's" or cardiff or "cardiff's" or newport or "newport's" or st asaph or "st asaph's" or st davids or swansea or "swansea's").ti,ab,in,ad.

17. (aberdeen or "aberdeen's" or dundee or "dundee's" or edinburgh or "edinburgh's" or glasgow or "glasgow's" or inverness or (perth not australia*) or ("perth's" not australia*) or stirling or "stirling's").ti,ab,in,ad.

18. (armagh or "armagh's" or belfast or "belfast's" or lisburn or "lisburn's" or londonderry or "londonderry's" or derry or "derry's" or newry or "newry's").ti,ab,in,ad.

19. or/11-18

20. (exp "arctic and antarctic"/ or exp oceanic regions/ or exp western hemisphere/ or exp africa/ or exp asia/ or exp "australia and new zealand"/) not (exp united kingdom/ or europe/)

21. 19 not 20

22. 10 and 21

Note: search strategy is inclusive of a tested and peer-reviewed UK filter to save time and resource costs when searching for research from UK settings (see Ayiku et al., 2019).

Reference

Ayiku, L., Levay, P., Hudson, T., Craven, J., Finnegan, A., Adams, R., & Barrett, E. (2019). The Embase UK filter: validation of a geographic search filter to retrieve research about the UK from OVID Embase. Health Information & Libraries Journal, 36(2), 121-133.

# Inclusion and Exclusion Criteria

|  | Inclusion criteria | | Exclusion criteria | | Justification | | |
| --- | --- | --- | --- | --- | --- | --- | --- |
| Publication Type | Original research articles | | Exclude; Systematic or other literature reviews, commentaries, letters, book chapters. | | We intend on synthesising peer-reviewed articles which evaluate PI activity within specific research projects; others forms of publication are beyond the scope of our review. | | |
| Broad topic | Public Involvement in research   - **Public involvement** in research means research that is done 'with' or 'by' the public. The public may relate to patients, carers, family members or other members of the public. - Terms such as patient involvement, co-production, advisory group, lay advisors, public contributors might also be used. | | Studies where the public are involved as ‘subjects’ or ‘participants’  Studies focused on research engagement e.g. where researchers aim to connect with or inform a community without partnership working,  Public Involvement in service evaluation/improvement or audit.  Public Involvement across an organisation (as opposed to PI within research studies) | | Exclusion criteria included to ensure the contextual factors and strategies we collate are relevant to involvement in research studies/projects i.e. there may be different approaches or contextual influences for involving members of the public in e.g. NHS service development or organisational level activity. | | |
| Focused topic | Public Involvement in applied health/social care research   - **Applied research** is solution focused, conducted to address a specific problem e.g. to improve mental health outcomes or reduce health inequality. | | Public Involvement in clinical trials/research (e.g. trials to test effectiveness of a new drug or treatment for patients)  Studies that focus on psychometric testing/development of outcome measures. | | Clinical research may introduce specific barriers and facilitators to involvement and shared decision-making that are beyond scope of the present review (e.g. complexity of information and legal and/or ethical processes for controlled drug trials etc.) | | |
| Geography | Research conducted in the UK | | Research conducted outside of the UK | | We intend on synthesising evidence generated within the context of the UK research landscape/PI guidance thus articles conducted outside of this context are beyond scope. | | |
| Population | Adults (18 years or older) | | Public contributors below 18 years of age. | | There may be distinctly different barriers to involvement for those under 18 in comparison to adult populations (e.g. for example, issues of parental consent, greater safety concerns and increased need for researchers to learn and adapt to more innovative forms of involvement (1-4). | | |
| *NOTES:* | ***If a paper includes multiple populations, it needs to be obvious which quotes or content is from the UK and/or an adult population. E.g., If the sample is 16-24, and the age is not specified within participant quotes etc. the paper will be excluded.*** | | | | | | |
| Design | | Evaluation studies that assess or make a judgement about e.g. the value or effectiveness of specific public involvement activity via systematic collection of data. Methods used may be:  Qualitative (words, quotes etc. as data) e.g.:   - Interviews, focus group discussions, open ended survey questions, reflective written feedback, observations.   Quantitative (numerical data) e.g.:   - Cohort studies - Cross-sectional surveys   Mixed methods:   - Includes both qualitative and quantitative components. | | Descriptive studies with no evaluation.  General reflections on public involvement activity with no indicated collection **and /or** analysis of data.  Evaluation of plans, frameworks, strategies or other that does not include consideration of actual public involvement activity. | | The present review intends to gain insight into effective strategies and hindering/facilitating contextual factors which are not clear from descriptive papers only.  General reflections can lack transparency regarding their conclusions.  Evaluations of plans and strategies etc., are beyond the scope of the present review. |  |

**References**

1. Rouncefield-Swales A, Harris J, Carter B, Bray L, Bewley T, Martin R. Children and young people’s contributions to public involvement and engagement activities in health-related research: A scoping review. PLoS One [Internet]. 2021 Jun 9;16(6):e0252774. Available from: https://doi.org/10.1371/journal.pone.0252774
2. Liabo K, Boddy K, Bortoli S, Irvine J, Boult H, Fredlund M, et al. Public involvement in health research: What does “good” look like in practice? Res Involv Engagem [Internet]. 2020 Mar 31 [cited 2022 Aug 15];6(1):1–12. Available from: <https://researchinvolvement.biomedcentral.com/articles/10.1186/s40900-020-0183-x>
3. Yamaguchi S, Tuong J, Tisdall EKM, Bentayeb N, Holtom A, Iyer SN, et al. “Youth as accessories”: Stakeholder Perspectives on Youth Participation in Mental Health Policymaking [Part II]. Adm Policy Ment Heal Ment Heal Serv Res. 2023;50(1):84–99.
4. Warraitch A, Lee M, Bruce D, Curran P, Khraisha Q, Wacker C, et al. An umbrella review of reviews on challenges to meaningful adolescent involvement in health research. Heal Expect. 2024;27(1).

# Examples of public involvement in the present review.

Table F1. Examples of how the present review was conducted in alignment with the NIHR UK Standards for Public Involvement (NIHR, 2019).

| **Standard** | **Brief definition of standard** | **Example application of standard** |
| --- | --- | --- |
| **Inclusive Opportunities** | Ensure public involvement partnerships are accessible and include a range of people and groups, as informed by community and research needs. | Engaging with regional networks to discuss the relevance and acceptability of review concept from a public perspective, prior to project/protocol development.  Flexible opportunities to contribute i.e. public partners identifying which stage(s) of the review they would like to/felt able to contribute to.  Offering remuneration for time and expenses in line with NIHR payment guidance. |
| **Working Together** | Work together in a way that values all contributions, and that builds and sustains mutually respectful and productive relationships. | Meeting with core public partners prior to protocol development, to develop shared understanding of roles, responsibilities and expectations (of all team members). |
| **Support and Learning** | Offer and promote support and learning opportunities that build confidence and skills for public involvement in research. | Development and delivery of bespoke training and guidance in line with public partner needs/requirements.  An identified main point of contact for public partners.  Pairing of public partner and research team members for intensive stages of involvement (i.e. during screening and selection process). |
| **Governance** | Involve the public in research management, regulation, leadership and decision making. | Public representation in team meetings for example to develop and finalise protocol detail.  Implementation of suggestions and recommendations from public partners during development of eligibility criteria. |
| **Communications** | Use plain language for well-timed and relevant communications, as part of involvement plans and activities. | Early commitment to using plain language wherever possible and offering additional support where this was not possible (i.e. when reviewing already published academic journal articles).  Co-authored dissemination materials. |
| **Impact** | Seek improvement by identifying and sharing the difference that public involvement makes to research. | Including co-authored reflections on the benefits, challenges and learning from public insight/experience within dissemination materials. |

# Quality assessment: Methodology of included studies - from Critical Appraisal Skills (CASP) Programme, 2023.

| Study | Was there a clear statement of **the aims**? | Is a qualitative **methodology** appropriate? | Was the **research design** appropriate to address the aims of the research? | Was the **recruitment strategy** appropriate to the aims of the research? | Was the **data collected** in a way that addressed the research issue? | Has the **relationship** between researchers and participants been adequately considered? | Have **ethical issues** been taken into consideration? | Was the **data analysis** sufficiently rigorous? | Is there a **clear statement** of findings? | How **valuable** is the research? |
| --- | --- | --- | --- | --- | --- | --- | --- | --- | --- | --- |
| Aabe et al., 2019 | Yes | Yes | Yes | Yes | Unsure | Unsure | Unsure | Unsure | Yes | Mid |
| Beighton et al., 2019 | Yes | Yes | Yes | Yes | Yes | Yes | Yes | Yes | Yes | High |
| Brett et al., 2022 | Yes | Yes | Yes | Yes | Yes | Yes | Yes | Yes | Yes | High |
| Buffel, 2019 | Yes | Yes | Unsure | Unsure | Unsure | Yes | Yes | Yes | Yes | Mid |
| Cotterel & Buffel, 2023 | Yes | Yes | Yes | Yes | Yes | Yes | Yes | Yes | Yes | High |
| Devonport et al. 2018 | Yes | Yes | Yes | Yes | Yes | Yes | Yes | Unsure | Unsure | Mid |
| Evans et al., 2022 | Yes | Yes | Yes | Unsure | Yes | Yes | Yes | Yes | Yes | High |
| Forbat et al., 2024 | Yes | Yes | Yes | Yes | Yes | Yes | Yes | Yes | Yes | High |
| Froggatt et al., 2016 | Yes | Yes | Yes | Yes | Yes | Yes | Yes | Yes | Yes | High |
| Lithander et al., 2023 | Yes | Yes | Yes | Yes | Yes | Unsure | Unsure | Unsure | Yes | Med |
| Litherland, 2018 | Yes | Yes | Yes | Unsure | Unsure | Yes | Unsure | No | Yes | Mid |
| McMenamin, 2021 | Yes | Yes | Yes | Yes | Yes | Yes | Yes | Unsure | Yes | High |
| Rowe, 2006 | Yes | Yes | Yes | Yes | Unsure | Yes | Yes | Yes | Yes | High |
| Slade et al., 2016 | Yes | Yes | Unsure | Unsure | Unsure | Yes | Yes | No | Yes | Mid |
| Stocker et al., 2021 | Yes | Yes | Yes | Yes | Unsure | Yes | Yes | Unsure | Yes | Mid |
| Sutton and Weiss, et al., 2008 | Yes | Yes | Yes | Unsure | Yes | Yes | Yes | Unsure | Yes | Mid |
| Thomas et al., 2021 | Yes | Yes | Yes | Yes | Yes | Yes | Unsure | Unsure | Yes | High |
| Willis et al., 2018 | Yes | Yes | Yes | Yes | Yes | Yes | Yes | Unsure | Yes | Mid |
| Worsley et al., 2022 | Yes | Yes | Yes | Yes | Yes | Yes | Yes | Yes | Yes | High |

# Quality assessment of public involvement reporting

Note: We amended the GRIPP2 topic areas into five questions as outlined in Table 1 below. The answers to these questions then facilitating judgments on the overall quality of PI reporting within the study (i.e. low, medium or high).

Table E1. Quality assessment of PI reporting as it relates to the original (evaluated) study, amended from the GRIPP2 short form (Staniszewska et al., 2017).

| Study | Has the **aim of PPI** been reported? | Is there a clear description of the **methods used for PPI** in the original study? | Have the **results/outcomes of PPI** in the original study been reported? (including both positive and negative effects) | Do authors comment on the **extent to which PPI influenced the original study** overall? (including both positive and negative effects) | Do authors **reflect/comment critically** on the orginal study (i.e. what went well and not so well) | Overall quality of reporting of PPI relating to the original (evaluated) study. |
| --- | --- | --- | --- | --- | --- | --- |
| Aabe et al., 2019 | Yes | Yes | Yes | Yes | Yes | High |
| Beighton et al., 2019 | Yes | Yes | Yes | Yes | Yes | High |
| Brett et al., 2022 | Yes | Yes | Yes | Yes | Yes | High |
| Buffel, 2019 | Yes | Yes | Yes | Yes | Yes | High |
| Cotterel & Buffel, 2023 | Yes | Yes | Yes | Yes | Yes | High |
| Devonport et al. 2018 | Yes | Yes | Yes | Unsure | Yes | Med |
| Evans et al., 2022 | Yes | Yes | Yes | Yes | Yes | High |
| Forbat et al., 2024 | Yes | Yes | Yes | Yes | Yes | High |
| Froggatt et al., 2016 | Yes | Yes | Yes | Yes | Yes | High |
| Lithander et al., 2023 | Yes | Yes | Yes | Yes | Yes | High |
| Litherland, 2018 | Yes | Yes | Yes | Yes | Yes | High |
| McMenamin, 2021 | Yes | Yes | Yes | Yes | Yes | High |
| Rowe, 2006 | Yes | Yes | Yes | Yes | Yes | High |
| Slade et al., 2016 | Yes | Yes | Yes | Yes | Yes | High |
| Stocker et al., 2021 | Yes | Yes | Yes | Yes | Yes | High |
| Sutton and Weiss, et al., 2008 | Yes | Yes | Yes, but minimal | Unsure | Yes | Med |
| Thomas et al., 2021 | Yes | Yes | Yes | Yes | Yes | High |
| Willis et al., 2018 | Yes | Unsure | Yes | Yes | Yes | Mid |
| Worsley et al., 2022 | Yes | Yes | Yes | Yes | Yes | High |

Table E2. Quality assessment of PPI reporting within the evaluation, amended from GRIPP 2 short form (Staniszewska et al., 2017).

| Study | Has the **aim of PPI** been reported? | Is there a clear description of the **methods used for PPI** in the evaluation? | Have the **results/outcomes of PPI** in the evaluation approach been reported? (including both positive and negative effects) | Do authors comment on the **extent to which PPI influenced the evaluation** overall? (including both positive and negative effects) | Do authors **reflect/comment critically** on the evaluation (i.e. what went well and not so well) | Overall quality of reporting of PPI within the evaluation |
| --- | --- | --- | --- | --- | --- | --- |
| Aabe et al., 2019 | Yes | Unsure | Unsure | No | No | Low |
| Beighton et al., 2019 | Unsure | No | No | Unsure | Yes | Low |
| Brett et al., 2022 | Unsure | Yes | Yes | Unsure | Yes | High/Med |
| Buffel, 2019 | Yes | Yes | Yes | Unsure | No | Med |
| Cotterel & Buffel, 2023 | Yes | Yes | Unsure | Yes | Yes | High/Med |
| Devonport et al. 2018 | Yes, but no patients | Yes | Not reported | No | No | Low |
| Evans et al., 2022 | Yes | Yes | Yes | Yes | Yes | High |
| Forbat et al., 2024 | Yes | Yes | Yes | Unsure | Unsure | Med |
| Froggatt et al., 2016 | Yes | Yes | Unsure | Unsure | No | Med |
| Lithander et al., 2023 | Yes | Yes | Unsure | Unsure | Yes | Med |
| Litherland, 2018 | Yes | Yes | Unsure | No | No | Med |
| McMenamin, 2021 | Unsure | Yes | Unsure | Unsure | Yes | Med |
| Rowe, 2006 | Not reported | None | Not reported | Not reported | No | Low – none reported |
| Slade et al., 2016 | Not reported | Not reported | Not reported | Not reported | No | Low – none reported |
| Stocker et al., 2021 | Not reported | Not reported | Not reported | Not reported | Yes | Low – none reported |
| Sutton and Weiss, et al., 2008 | Not reported | Not reported | Not reported | Not reported | No | Low – none reported |
| Thomas et al., 2021 | Unsure | No | No | No | Yes | Low |
| Willis et al., 2018 | No | Yes, but minimal | No | Yes, but minimal | Unsure | Low |
| Worsley et al., 2022 | Unsure | Yes, but minimal | Unsure | Yes, but minimal | Yes | Med |

Note: there were some challenges in determining whether authors were referring to the original study or the evaluation when referring to ‘the study’ e.g. in discussion sections.

# Additional Key Excerpts

Table F1. Example extracts for each theme/sub-theme within the review.

| **Theme** | **Sub-theme/codes** | **Example Excerpt** |
| --- | --- | --- |
| **Contextual factors** | | |
| Structural | Lack of acknowledgement around the reality of co-production (B) | “Despite all the rhetoric around NIHR for public engagement, and you get to write about this on one of the boxes on the forms, I think the system’s commitment to an authentic messy public engagement is not there yet. If your public engagement gets you to a pristine application that looks like it was written by a bunch of academics, that’s what they want but ours didn’t turn out like that” (Researcher) - Worsley et al., 2022 |
|  | Available funding for involvement (M) | “Paradoxically, if you try and do the right thing by people in terms of paying them and you’ve only got a limited budget, the amount of times you can come together is limited.” (Researcher) - Worsley et al., 2022 |
|  | Funder imposed time constraints (B) | ‘The longevity of the project was crucial to building trust and allowing a shared understanding of the research. However, commenting at the end of the study, Rachel asked ‘what's the next project, we are ready to start now!’, suggesting she felt familiar with the processes and structures of research at the end in a way the academics had not paid sufficient attention to at the beginning. This highlights a tension between the time-limited nature of most research projects and funder's timescales, and the importance of time to create the conditions for truly engaged research’ (Author text). -Thomas et al., 2021 |
|  | Funder feedback and outcomes (B) | ‘…although the likelihood of rejection was talked about often in the group, public advisors were disheartened by the feedback from [the funder]: *“That was disappointing. There was a hell of a lot of effort. I actually wrote about a 100- page proposal on my ideas.”* (Public partner)’. - Worsley et al., 2022 |
| Organisational | Clear commitment to collaboration (F) | “When I’d done my interview [a public partner] was on the panel. So, from my very first interaction with the project team, there’s [PI] included within that, and each member of the panel had their own set of questions and [the public partner’s questions] were  all asking about [PI] views and experience of that, so that was kind of my first introduction as part of this project.” (Researcher) - Forbat et al., 2024 |
|  | Embedded research support | ‘Facilitating context factors from the researchers perspectives were…feeling well supported by research colleagues in PI activites (definitely 75%, n=17) (Author text)’ – Brett et al., 2022 |
|  | Proximity to communities of interest (M) | "Minor hindering context factors included geographical distance between the [public partners] and the research team (mainly based in Belfast, Leeds, Oxford and Southampton) and therefore travelling inconvenience and lack of knowledge to contribute to certain areas of the research.” (Author text) - Brett et al., 2022 |
|  | Budget constraints (B) | “As true coproduction takes time, the process felt rushed towards  the end. This was due, in part, to the way in which public advisors were funded: It didn't end up being as good as it could have been because we just simply didn't have the time to make it so … Time constraints and if you like the capacity of our public advisors to maintain that track towards because of the way that they were funded to enable this process.” (Researcher) - Worsley et al., 2022 |
|  | Staff time and resource for PI activity (M) | ‘Whilst the presence of [public partners] during fieldwork visit was a support for the residents and the researcher, it did require that researchers paid attention to the activities undertaken by [public partners]. Alongside their own work, undertaking the interviews, this added another level of complexity in an already busy environment.’ (Author text) - Froggatt et al., 2015  “..expectations and resources, there weren't resources to provide the level of involvement for yourselves that we want, and the national standards advocate” (Researcher) – Evans et al., 2022 |
| Group/Interpersonal | Establishing good working relationships (F) | ‘We were very diverse in our way with different issues and different problems and different perspectives but maybe we were lucky but we operated brilliantly as a group…The dynamics were very good. We had a good team and I think that is absolutely critical.” (Public partner) - Brett et al., 2022 |
|  | Willingness to share decision making/poor management of power dynamics (M) | ‘[Public partner] echoed the point that they did not want to ‘offend’ [the researcher] when asked to review the interview guide despite being encouraged to do so. This internalisation of hierarchy and power is a major challenge to truly collaborative co-research’. (Author text)– Cotterell & Buffel, 2023. |
|  | Trust and mutual respect (F) | ‘We relied on each other's insider expertise and knowledge of research and Somali culture to negotiate the phases of research. In particular, informed consent was challenging for [a public partner] to explain to participants, requiring time and patience. In the data collection stage, through the process of bilingual co-interviewing, which required openness, trust and continual reflection, our relationship as co-researchers was cemented’. (Researcher) - Aabe et al., 2019 |
|  | Access to existing groups (F) | ‘One group, ResearchNet, comprised of adults with intellectual disabilities and had been facilitated by two healthcare professionals for a number of years. One of the researchers had also worked with the group over this time and had built up a relationship with them, negating the need to build up trust prior to commencing the study. (Author text)’ - Beighton et al., 2019 |
|  | Clear leadership (F) | ‘With time, commitment and skilled chairing of [the public partner group] relationships developed and the process of involvement became less confrontational and more  settled.’ (Author text) - Slade et al., 2016 |
|  | Conflicting opinions and priorities (B) | ‘Decision-making within the group was difficult at times, and consensus was not always reached.’ (Author text) – Rowe, 2006 |
|  | Language barriers and cultural differences (B) | ‘[The researcher] noted that because she could not speak Somali, it was challenging to develop trust and rapport with participants: “their non‐verbal cues were not always easy for me to read and this maintained my sense of being an outsider. I relied on [a public partner] to maintain rapport which felt uncomfortable at times.” (Author text) - Aabe et al., 2019 |
|  | Existing professional tensions (B) | “Both patients and practitioners reported to me, privately, that they wanted to raise some issues but they had felt unable to do so because the [advisory group] was being led by ‘experts from the University’. Some felt they had been ‘steered’ in a particular way to address a pre-set list of questions. Knowing the extent to which the academic partners tried to genuinely involve the patients and practitioner in the research process these comments felt very frustrating.” (Researcher) - Devonport et al., 2018 |
|  | Concerns around representation vs. professionalisation (B) | ‘..the danger of those involved in research becoming an uncritically officialized ‘community’ voice is well known and also risks homogenizing the experiences of what are in reality, likely to be diverse experiences and perspectives.’ (Author text) - Thomas et al., 2021 |
| Individual | Previous experience of involvement (F) | “The more of this sort of work you do, the better you get it actually challenging researchers, because it takes a lot of confidence to do that, when you start off you're not very confident.” (Public partner ) - Evans et al., 2022  ‘…researchers who had limited experience in [public involvement] reported that they lacked confidence in knowing how to involve [public partners] and how to use the feedback they provided’ (Author text). -Lithander et al., 2023 |
|  | Passion for change/personal importance of the topic (F) | ;Public [partners] reflections around motivation were dominated by efforts to change the mental health system, not just for oneself but also for others: ”Let’s do this. Let’s do it for Liverpool. Let’s do it for everybody, the whole nation. We’ve come up with  something for improving mental health. It’s that sort  of attitude.” – (Public partner) – Worsley et al., 2022 |
|  | Conflict resolution skills (M) | ‘…attempts to progress the discussion may have been perceived as researchers closing down conversations on particular topics.Indeed, some public advisors thought that the researchers were trying to steer the meetings in a certain direction: “There were some who were just a bit nasty who thought the academics had their own agenda.” (Public partner) – Worsley et al., 2022 |
|  | Personal circumstances and/or trauma (B) | ‘Following completion of the three mandatory training sessions, four male and four female co- researchers decided not to participate any further in the research project. This was due to their personal circumstances, health issues, or having other commitments’.(Author text) – Cotterell & Buffel, 2023 |
|  | Confidence to contribute (F) | “I had little experience of research when this journey began and needed to assert myself in these early meetings. I was glad that the problems facing our community were being taken seriously by the researchers but as we discussed the research question, I kept stating that the focus should be on families’ access to services. The other members of the team agreed and together we planned an interview schedule that would explore this issue.” (Public partner) - Aabe et al., 2019 |
|  | Researcher interpersonal skills and knowledge (M) | “I think maybe the staff, certainly in our stream, could have had a one day workshop on user involvement … I don’t think my team at least knew how to deal with the [public partners]” (Researcher) - Brett et al., 2022  “[The lead researcher] was always affirming and saying “Thank you,” but she didn’t let [group members] go on too long, if I felt they were going on a bit long, she would bring them […] just gently say, “Oh, thank you so much, that was really helpful.” She was incredibly affirming which actually gave…even made me feel relaxed, you know, to say something that I might want to say, you felt you could contribute to the meeting…” (Public partner) (Author text) – Forbat et al., 2024 |
|  | Local knowledge (F) | “Also, like myself, when you live in an area you get a feel for it, you have an intuitive knowledge about the place: you read local papers and get local magazines, see notices, recognize faces, you know the place... All this helps when interviewing and trying to understand [participants’] perspective.” (Public partner) – Buffel, 2019 |
|  | Academic language and methods (B) | “Sometimes the documents can be very complex. And I mean the challenges I found were the, you know, the theories behind things, and I think I was a confused critical realist at one point, you know. I'm just absolutely, totally gobsmacked at how  [complex] things are.” (Public partner) – Forbet et al., 2024 |
|  | Concerns around time contribution (B) | “I think one of the difficulties is that all the other members of the research team are full time researchers and working on the project and it's quite difficult with the [public partners] - you feel quite conscious that you are taking up someone else's time when they could be doing something else.” (Researcher)- Brett et al., 2022 |
| **Effective PI strategies** | | |
| Principle-based strategies | Pre-identifying and planning principles | ‘Pre-identified principles of supportive involvement for  people with dementia were adopted in meetings (Litherland & Capstick, 2014) including building trust, meeting regularly (and keeping contact between meetings), providing supportive environments, having sufficient time and being flexible, adapting written materials and providing cues and prompts.’ (Author text) - Litherland et al., 2018 |
|  | Flexibility of methods | ‘These less formal opportunities were invaluable in developing trust, breaking down unhelpful them/us oppositions and developing shared understanding of the research endeavour while holding differences in perspective respectfully within it.’ (Author text) – Slade et al., 2016 |
|  | Engaging in two-way dialogue | That’s where it comes down to relationships as well, sometimes these researchers are just like an anonymous person. On this project they’re not. (Carer) – Litherland et al., 2018 |
|  | Embedding of emotional support | ‘[Public involvement] process was enhanced by group sessions with the project leader who provided emotional and practical support throughout phase one.’ (Author text)– Willis et al. 2018 |
|  | Co-production/Shared power approach | ‘[Public partner] described how being involved from the outset, before the key focus of research had been determined enabled her to feel more attuned to the origin and importance of the research topic. Members of the friendship research group described the nature of decision-making together as being non-linear but “like a shoal of fish”, travelling along together then responding to a change of direction “coming from within the group”. [Public partner] contrasted this form of direction from within to “feeling on the outside” in many of her other non-participatory experiences of research’ (Author text) – McMenamin et al., 2021 |
|  | Building in reflection opportunities | 'After each monthly meeting, [public partners] were invited to a debrief where further thoughts, reflections and questions could be raised with a member of the academic team. (Author text)' - Forbat et al., 2024 |
|  | Embedding involvement throughout | ‘Public contributors played an active role in the process of planning and implementing public involvement throughout the study. They decided how the involvement processes, summarized in the research application, were undertaken in practice. They held discussions at Public Involvement team meetings and undertook an annual Public Involvement review that initiated additional activities to extend the role of public contributors and strengthen the practice’. (Author text) – Evans et al., 2022  “I was impressed at how much involvement there was in all the different stages of the project. So, it certainly wasn't just like in a tick box.” (Public Partner) – Evans et al., 2022 |
|  | A focus on accessibility | “I enjoyed the way that this [session] has been presented, because I think that having all of us in roles [for the role play session] is useful. Because being in a role places yourself in that mindset, and it's all about the feeling. And it IS about the feeling, it's not about the operation. I think I'd have been really flooded with information if I'd had the full transcript. So having quotes that looked at a particular aspect, was really useful to allow me to focus on that.” (Public partner) – Stocker et al., 2021 |
| Practical strategies to involve the public | Group meetings and/or workshops | ‘A key element of [embedding involvement] was involving the [public partners] in regular study team meetings, which both facilitated involvement and helped to build relationships’. (Author text) - Brett et al., 2022 |
|  | Online activity | “The research team have created accessible and flexible opportunities for me to be involved, for example I have been able to share my thoughts over Zoom.” (Public partner) – Lithander et al., 2023 |
|  | Role-play activity | ‘Role-play was viewed as a useful way of stepping into the mindset of the interviewee and understanding. None of the attendees expressed or showed signs of reluctance to take part’. (Author text) - Stocker et al., 2021 |
|  | Relationship building activity | ‘Meetings are not rushed, and usually begin with an informal lunch where everyone can relax and catch up with each other.’ (Author text) – Litherland et al., 2018 |
|  | Gatekeeper involvement | [When discussing factors supporting co-production] ‘The involvement of at least one person who is willing and able to advocate for a community group and to bridge the gap between research institutions and community organization.’ (Author text) - Aabe et al., 2019 |
|  | Peer facilitators | “[The Chair] was absolutely a central point and … it’s important to have that - somebody who is able to facilitate and involve others, not just himself, from a wider group” (Researcher) - Brett et al., 2022 |
|  | Early involvement and expectation setting. | “[Public partners] were embedded from the start so it just kind of became second nature almost. So, in terms of every time we have one of our monthly investigator meetings, there is always a section on the agenda for an update from the user advisory group and every time if we’re writing a paper we’ve always involved [the Chair] as a co-author because he’s a co-investigator on the study. So it’s just sort of always been there~. (Researcher) - Brett et al., 2022 |
|  | Stakeholder events | ‘Stakeholder workshops—proposed role, to support public members at the events, was extended. Additional roles included recruiting public members, co‐planning the agenda and room layout to address public needs, facilitating discussion groups and  co‐presenting’. (Author text) – Evans et al., 2022 |
|  | Remuneration | “We have done a lot of work. We are paid for our role following INVOLVE user involvement rates – this recognises and values our contributions.” {Public co-authors]- Litherland et al., 2018 |
|  | Establishing clear aims and documentation | ‘The identification of clear roles and activities ensured that [public partners] felt a part of the project team.(Author text) - Froggatt et al. 2015 |
|  | Integrated communication and feedback channels | ‘The research team have been very positive about our work, and they tell us what has changed because of our involvement. It is important to know that our contributions are making a difference and it is worth our efforts.’ [Public co-authors] - Litherland et al.,2018 |
|  | Distinct allocation of time and resources for PI | ‘Various mechanisms that helped foster and support the integration and engagement of [public partners] within the study were identified, including […], allocating time and resources to [public involvement group].’ (Author text) – Brett et al., 2022 |
|  | Training | ‘[Public partners] completed three mandatory interactive research training sessions prior to conducting interviews. These sessions aimed to enhance and share knowledge between co-researchers about research processes including ethical practice, safeguarding, and qualitative interviewing techniques.’ (Author text) – Cotterel & Buffel, 2023 |
| **Outcomes** | | |
| Organisational impact | Culture Change | ‘One respondent said that he now advocated for public involvement in other studies he was working on because he valued this effect on team outlook.’ (Author text) - Evans et al., 2022 |
|  | Changes to service delivery | ‘The [public partner] since reported that the LGBTQ+ organisation now hosts regular events at the weekends to alleviate loneliness.’ (Author text) - Cotterell & Buffel, 2023. |
|  | Increased public representation | ‘[Public partner 1] spoke at a national health inequalities conference on the role the community partners were playing in shaping the research, whilst [public partner 2] co-led a workshop at an international clinical conference on the lived experiences of low-income patients attending GP consultations for mental health’ (Author text) -Thomas et al., 2021. |
| On the research process | Improved patient-facing materials | ‘[Researchers] listed several benefits of [public partner] input including ensuring that patient‐facing documents contained clear information in plain language, that terminology was appropriate for the target audience, and that the study procedures considered the needs of [people with Parkinsons]. Involvement of the [public partners] was deemed to have led to tangible changes to the study documentation…’ (Author text) - Lithander et al., 2023 |
|  | Ensuring patient-centred/focused approach | “[Public partners] made the case for culture and ethnicity to be a focus of the research, and this was implemented through extending the study in a number of ways, improving its design and creating a closer match with the community’s needs and interests.” (Researcher) - Slade et al., 2016 |
|  | Increased engagement with marginalised groups | “I don’t think the very elderly people would have disclosed as much to students or young academics, as they were often ashamed of their problems such as fear of computers, severe deafness, using a commode...”(Public partner).- Buffel, 2019  ‘…while it was acknowledged to be difficult to quantify the effect on study recruitment, these changes [to study documentation] were thought to have improved the quality of the research and were anticipated to positively impact on research participant  recruitment’ (Author text) – Lithander et al., 2023 |
|  | Increased relevance of research project | “They’ve been involved in so many different aspects of it, in terms of giving feedback on results, and I know they have done a lot of stuff on the qualitative work streams […] identifying themes and going through comments. I think, just making sure that what we’re producing is actually relevant to men [the target population] is the main thing.” (Researcher) - Brett et al., 2022 |
|  | Influence on data interpretation and findings | ‘Our themes and theme descriptors were reviewed through the new lens, and our data interrogated for other examples of issues brought up by PPI partners, such as differing levels of respect for health-care staff skills, and the role of resident choice in their GP care. We also integrated additional ideas and concepts into the analysis framework for our wider data set.’ (Author text) - Stocker et al., 2021 |
|  | Inclusion of patient focus in dissemination materials | ‘In the final meeting, the group made comments on the draft final report, particularly advocating a stronger message directed towards politicians, and gave several suggestions as to where the report should be disseminated.’ (Author text) - Sutton and Weiss, 2008 |
|  | Greater insight into ethical issues | ‘ "We got really helpful feedback from [PPIE members] about the need to make it more accessible, so kind of adapted the approach, and from that came up with this idea of guardian angels." [...] the agreement that PPIE members act as ‘guardian angels’ to call out academics if they veered into technical or complex descriptions [during study workshops] that [participants] might experience as exclusionary’. (Author text) - Forbat et al., 2024 |
|  | Changes to project PI approaches | ‘[Public partners] played an active role in the process of planning and implementing public involvement throughout the study. They decided how the involvement processes, summarized in the research application, were undertaken in practice. They held discussions at Public Involvement team meetings and undertook an annual Public Involvement review that initiated additional activities to extend the role of [public partners] and strengthen the practice”. (Author text) - Evans et al., 2022 |
| Public partner impact | Self-confidence and social capital | “Since I’ve been in with this research and the people, I’ve felt as though I can open up and I could talk. They know more about me and that’s given me trust to be able to talk.” (Public partner) – Worsley et al., 2022 |
|  | Skill development | “The training we received gave confidence to every one of us who was to embark on the project. During the training, I improved my listening, my communication and analysis skills. I see myself already using these skills in my other areas of work.” (Public partner) Buffel, 2019 |
|  | Ability to share knowledge with the community | ‘ [Public partner] gave an example where he had asked the coordinator of a large LGBTQ+ group in the city centre to host an event focusing on loneliness in older LGBTQ+ people on the weekend. This was because several interviewees had reported that the weekend was when they felt most lonely.’ (Author text) – Cotterell & Buffel, 2023 |
|  | Access to further education or employment | ‘As a result of becoming involved with this project a number of the [public partners] have taken up further courses and others have been trained as ‘Additional Learner Support’ workers for the college.’ (Author text) – Rowe, 2006 |
|  | Further research involvement | ‘The project also stimulated the coresearchers’ interest in undertaking additional research projects. These have included: participation in research on the role of the  environment in contributing to health and well-being in old age; a project using visual art and heritage practices to imaging an “age-friendly community for all ages”; and a study focused on preventing social isolation’. 9Author text) – Buffel, 2019 |
| Researcher impact | Greater understanding of topic area | ‘Meeting and hearing about the experiences of the participants in the survey resulted in considerable reflection and learning for the researchers. Being exposed to many different ways of life had mostly led them to re-evaluate their own assumptions.’ (Author text) - Rowe, 2006 |
|  | Greater ability to engage in PI activity | “This is my first experience of research, having come from a clinical background I will take these experiences, and the wider experience of trying to involve service users meaningfully with me in my future research posts.” (Researcher) - Slade et al., 2016 |
|  | Increased rapport with public partners | “You are always at risk of a certain type of tokenism with patient engagement activities and on this occasion there wasn’t any. It was a very real and productive way of adding value to the project as a whole I thought. It made it more ‘real’ to all of us” (Researcher) - Brett et al., 2022 |
| Discrete products (of involvement) | Recommendations to improve PI in research | ‘The findings also had an academic impact, addressing several gaps in knowledge within the research community including creating a co-produced guide on facilitating co-research in minoritised populations (Cotterell et al. 2022). This guide was disseminated by [public partners] to local community organisations to promote the inclusion of older minority groups in research and practice.’ (Author text) – Cotterell & Buffel, 2023. |
|  | Educational or other tool(s) | “I had a meeting with 4 [public partners] (and email contact with one other) to help develop INSPIRE [a measure of recovery support]. This proved extremely useful and I appreciated the time and energy [public partners] gave to this.” (Researcher) - Slade et al., 2016 |
|  | Training package(s) | “We asked [public partners] to design and run a half day training course on interviewing people with psychosis for our trial research teams, which was extremely valuable and in hindsight, I wish we’d done it earlier.” (Researcher) – Slade et al., 2016 |
| **Public partner experiences** | | |
| Experiences of meaningful involvement | Authentic involvement | “I genuinely felt, and I’ve said this to various people, but this wasn’t just a tick box exercise, ooh yes, I’ve consulted carers, it was a genuine ... let’s see how you can get involved and I’d like to incorporate your ideas in it, so it did feel like genuine involvement which was great.” (Public partner) - Beighton et al., 2019 |
|  | Feeling valued and supported | “Right from day one I felt I was involved and included and I attended the first meeting where everyone attended at [cit] and I received a very warm welcome. People came and introduced themselves to me and I just felt welcomed and valued” (Public Partner) - Brett et al., 2022 |
|  | Seeing and contributing to change | ‘Reflecting on their involvement in the study, [public partner 1 and 2] felt that it was their growing sense of the project's potential to make an impact on practice and policy that sustained their motivation, *“I knew it was an important subject […] the thing that would’ve really put me off was if it was just research*” (Public partner) - Thomas et al., 2021 |
|  | Opportunity to share and learn | “I value and enjoy working together with older people, we learn a lot from each other. Together we can influence the planning and development of services…. I welcomed  the opportunity to learn from others in this project, and to learn new skills, and to have my contributions valued” (Public partner). - Buffel, 2019 |
|  | Feeling heard and included | ‘We, we are actually being listened to and taken note of. Our opinions counted taken seriously.” (Public partner) -Beighton et al., 2019 |
|  | Mutual respect | ‘Public contributors acknowledged how their sense of worth within the study team increased through involvement. This also nurtured their motivation and enthusiasm, further building the team bond and sense of shared purpose. Their sense of self‐worth was also enhanced by the respect given to their contributions and the atmosphere of mutual respect within the team.’ (Author text) - Evans et al., 2022 |
|  | Sense of ownership | ‘Having control and being given the reins . . . not about consultation it’s about us steering it.’ (Public partner) – McMenamin et al., 2021 |
|  | Camaraderie | “I think it’s great to see the seven of us . . . we can talk, you can talk about different things and we’re just ordinary people at that stage” (Public partner) - McMenamin et al., 2021 |
|  | Giving back to the community | “I felt that I was giving these women [in the community] information that they would not have got for themselves. (Public partner).” – Rowe, 2006 |
|  | Feeling connected | ‘Both groups commented that it was not just the purposeful, creative thinking but the fun and laughter that cemented their commitment to the group and the research.’ (Author text) - McMenamin et al., 2021 |
| Unintended harms | **Experiencing negative attitudes** | |
|  | Hearing or experiencing negative discrimination | “[Public partners] reported difficulties in maintaining a neutral stance when particular views were expressed. Christine (70 years old), for example, talked about one of her interviewees expressing racist views, commenting: “Shocking! I really  had to bite my tongue.” (Public partner) – Buffel, 2019 |
|  | Avoidance from professionals | “We couldn’t meet with all staff together formally. We quickly realised that, that didn’t work because all the staff were otherwise engaged . . . There were some people who didn’t take part. There was an admin worker who I thought actively avoided it—she was late, some work crisis or sick.” (Public partner) - Willis et al., 2018 |
|  | **Emotional burden of involvement** |  |
|  | Re-traumatisation | ‘Challenging relationships with co-facilitators and also with the management at times felt like a re- experience of earlier trauma from growing up with confusion’. (Author text) - Willis et al., 2018 |
|  | Emotional burden of sensitive topics | “One thing I didn’t anticipate was how much this experience touched on (my) painful memories of homophobia. For me, it was about being mindful ofthat personal impact. Looking after myself... The project leader is a massive support, really. You can speak to her about most things”(Public partner) - Willis et al., 2018 |
|  | Anxiety and negative anticipation | '...advisory sessions could be anxiety-provoking for [public partners], especially when needing to challenge directly some staff members’ deep-seated beliefs' (Author text) - Willis et al., 2018 |
|  | **Frustration and disappointment** | |
|  | Perceived lack of importance of PI | “I feel moved to express my amazement at the absence of service user involvement in the development and design of a study about an issue that is so fundamentally about a personal experience and a personal and individual journey. Whilst I do acknowledge efforts at getting service user/researchers involved, it is too late in the day.” [Prospective public partner who declined to be involved] - Slade et al., 2016 |
|  | Frustration with timescales | “The clock is ticking and I want things to be happening quicker than perhaps they can happen.” (Public partner with dementia) – Litherland et al., 2018  “Yes, more time is needed. Definitely. On a project looking at loneliness in ethnic minority communities? Of course. I could find ladies who never go out, don’t speak any English and struggle with everything they do. Different strategies could be used . . . but it was a rush and I couldn’t do that, sorry.” (Public partner) Cotterell & Buffel, 2023 |
|  | Lack of feedback on impact of contribution | “I think feedback was pretty good but I think it could have been better. I think it could have been built in as kind of a requirement in a sense […] Give us a bit of feedback and maybe how we could do it differently next time etc …” (Public partner) - Brett et al., 2022 |
|  | Research design constraints | “Although the research team had some sympathy towards our viewpoint, they chose to focus on the positivist tradition of research as they felt it generated data that was more valid and the research would be more respected and meaningful. There was much discussion and researchers listened at the start, but then focused very much on the approach they wished to take.” (Public partner) - Slade et al., 2016 |
|  | Lack of involvement opportunity over time | “I feel some sadness at my own lack of involvement with the [advisory] group – possibly through the changes in my own life rather than through the project”. (Public partner) – Slade et al., 2018 |
|  | Mismatched expectations | ‘[Public partners] commented that the priorities of the researchers sometimes lost their patient focus, and it was the role of the [public partners] to bring this back. Researchers recognised that sometimes there was a mismatch between their focus and the focus of the [Public partners]’ (Author text) - Brett et al., 2022 |
|  | **Further marginalisation** | |
|  | Overly form, impersonal involvement | ‘ [A public partner] who had not previously met the other community members, for example, explained how she felt quite isolated at early meetings because “we were there as community partners but we as a "community" didn't know each other.” (Public partner) - Thomas et al., 2021 |
|  | Lack of clarity and feeling unprepared | “[Training] gave a good grounding for the role but I still felt ‘thrown in at the deep end’ when it came to making appointments and interviewing people.” (Public partner) - Rowe, 2006 |
|  | High level of researcher control | “There was one lady that was really dominant and was better educated than myself but she thought all of the academics were taking over.” (Public partner) - Worsely et al., 2022 |
|  | Lack of power and tokenism | ‘In the initial stages of the project, community partners felt that ‘we were probably defined by what we do rather than who we are’ (ie resident or researcher), and that this impacted negatively on them when they felt they did not bring ‘professional’ expertise to the table’. (Author text) - Thomas et al., 2021 |
